# Coseismic crustal seismic velocity changes associated with the 2024 M_W_ 7.5 Noto earthquake, Japan

**DOI:** 10.1186/s40623-025-02177-x

**Published:** 2025-04-21

**Authors:** Nicolas Paris, Yuji Itoh, Florent Brenguier, Qing-Yu Wang, Yixiao Sheng, Tomomi Okada, Naoki Uchida, Quentin Higueret, Ryota Takagi, Shin’ichi Sakai, Satoshi Hirahara, Shuutoku Kimura

**Affiliations:** 1 Université Grenoble Alpes, CNRS, INRAE, IRD, Grenoble INP, IGE, Grenoble, France; 2 Earthquake Research Institute, The University of Tokyo, Tokyo, Japan; 3 ISTerre, Université Grenoble Alpes, Grenoble, France; 4 ITES UMR 7063, Université de Strasbourg/CNRS, Strasbourg, France; 5 Laboratory of Seismology and Physics of the Earth’s Interior, School of Earth and Space Sciences, University of Science and Technology of China, Hefei, China; 6 Research Center for Prediction of Earthquakes and Volcanic Eruptions, Graduate School of Science, Tohoku University, Sendai, Japan

**Keywords:** 2024 Noto earthquake, Seismic velocity change, Ambient noise seismic interferometry, Static stress, Dynamic stress

## Abstract

The 2024 $\hbox {M}_{\textrm{w}}$ 7.5 Noto earthquake, Japan, was preceded by an intense seismic swarm thought to be driven by upward fluid migration. Crustal seismic velocities vary with external perturbations caused by earthquakes, and the presence of pressurized fluids in the crust amplifies the resulting coseismic velocity change. Hence, we characterize subsurface fluid by measuring the coseismic velocity change associated with the 2024 mainshock. For this purpose, we perform multi-frequency-band ambient noise seismic interferometry using data from permanent and temporary seismic stations. Significant coseismic velocity drops are observed, with an average decrease of about 0.5% inside the Noto peninsula, reaching 0.6–0.8% in the regions near the coseismic slip peaks. The observed velocity drops inside the peninsula correlate well with the modeled static-stress-change-induced velocity drops and peak ground velocity (PGV) and acceleration (PGA) as proxies of dynamic stress change. However, their respective contribution to the observed coseismic velocity drop remains unclear because of the similarities in their spatial pattern. Outside the Noto Peninsula, the observed velocity drops average around 0.1%, which is predominantly attributed to dynamic stress changes from passing waves because modeled static stress changes are negligible at these great distances. Although the addition of temporary stations significantly increases the resolution of the velocity drop measurements in the pre-mainshock swarm zone, our results exhibit no large velocity drop anomaly in this region, suggesting that the amount of pressurized fluids in the shallow crust down to $\sim$ 2.5 km depth is not anomalously large. This implies that the upward migration of fluids preceding the mainshock is likely confined to greater depths.

**Graphical Abstract:**

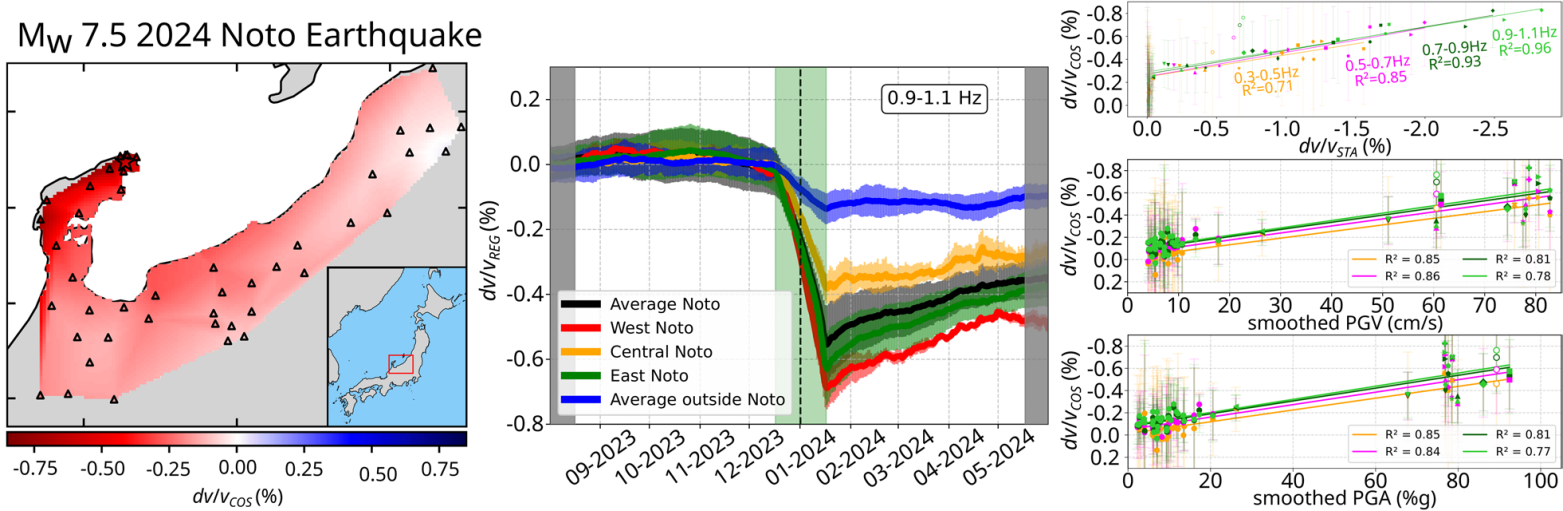

**Supplementary Information:**

The online version contains supplementary material available at 10.1186/s40623-025-02177-x.

## Introduction

**Fig. 1:**
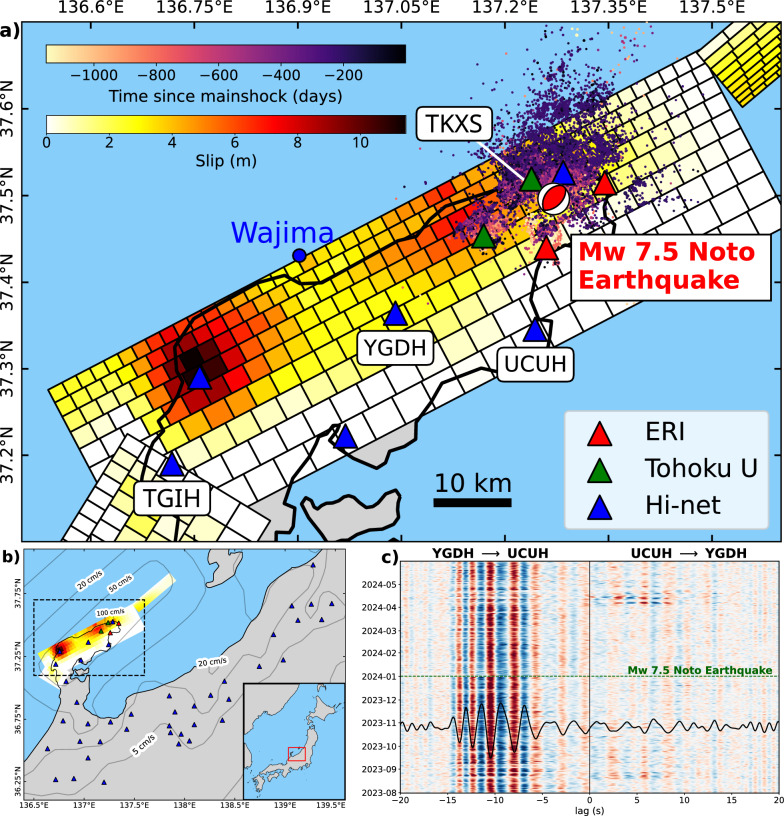
**a** Tectonic setting. The colored patches are the coseismic slip model of the 2024 Noto earthquake (Ma et al. 2024). Colored dots are relocated hypocenters of regional seismicity down to 15 km depth before the 2024 mainshock, color-coded with time (Yoshida et al. 2024). The beach ball indicates the mainshock location and its moment tensor from the Japan Meteorological Agency and F-net, respectively. Triangles indicate the location of seismograms used in this study, with colors indicating different observers as labeled. The blue dot indicates Wajima station for sea wave height measurement. **b** Map of sites used in this study. Grey contours represent the PGV calculated by USGS. Triangles mark the selected stations approximately within the 5 cm/s PGV contour lines. The black dashed box indicates the area in **a**. **c** Correlogram between vertical component seismograms from sites YGDH and UCUH, filtered between 0.3 and 1.1 Hz. The black waveform represents the average daily correlation between 1 August 2023 and 1 June 2024. The green dashed lines indicate the time of the 2024 Noto earthquake mainshock

On 1 January 2024, a $\hbox {M}_{\textrm{w}}$ 7.5 earthquake occurred along the northern coast of the Noto Peninsula, Japan (Fig. 1a, b). The total rupture zone spans more than 150 km, extending toward the northeast offshore area and southwest along the northern coast of the peninsula (Fujii and Satake 2024; Ma et al. 2024; Okuwaki et al. 2024). This earthquake was preceded by a long-lasting seismic swarm that began in late 2020, with intermittent seismicity in the same region as the mainshock epicenter (Amezawa et al. 2023; Yoshida et al. 2023, 2024; Kato 2024). The swarm seismicity migrated from $\sim$ 15 km to $\sim$ 5 to 10 km depth over time (Amezawa et al. 2023). Previous studies suggest that this swarm migration is caused by the diffusion of mantle-origin fluids from the lower crust to the shallower depth (Nishimura et al. 2023; Amezawa et al. 2023; Okada et al. 2024a; Wang et al. 2024; Takano et al. 2024; Umeda et al. 2009; Nakajima 2022; Umeda et al. 2024). These observations pose a simple question of whether this upwelling fluid flow accumulated anomalous amounts of fluids at the subsurface by the time of the 2024 Noto earthquake. This study primarily aims to investigate the presence of anomalous amounts of fluids in the subsurface of the Noto Peninsula, down to a depth of 2.5 km.

Earthquakes impact seismic velocities in the surrounding medium by various processes such as damage at shallow depths due to coseismic dynamic stress perturbation (Wang et al. 2019; Sheng et al. 2021), static stress change (Nishimura et al. 2000; Wang et al. 2019; Sheng et al. 2022), and pore pressure changes induced by triggered fluid migration (Wang et al. 2021). Particularly, the susceptibility of seismic velocities to seismic shaking can reveal areas with anomalous conditions, such as high crustal fluid pressure (Brenguier et al. 2014). Therefore, the 2024 Noto earthquake offers a unique opportunity to explore the presence of fluids in the subsurface by measuring the regional seismic velocity changes. To measure velocity perturbations induced by earthquakes, velocity measurements before, during, and after the earthquake are required, which can be achieved using ambient noise seismic interferometry. Ambient noise seismic interferometry relies on the correlation of seismograms to retrieve an approximation of the Green’s function between the stations. Following the successful retrieval of Green’s function using diffusive seismic coda waves (Campillo and Paul 2003) and its subsequent application to ambient seismic noise (Shapiro and Campillo 2004), the seismic wave correlation technique has been used extensively to measure velocity perturbations in various settings over the last two decades (Brenguier et al. 2008; Takagi et al. 2012; Brenguier et al. 2014; Sheng et al. 2022; Çubuk Sabuncu et al. 2024).

In this study, we measure coseismic relative seismic velocity perturbations caused by the $\hbox {M}_{\textrm{w}}$ 7.5 Noto earthquake in multiple frequency bands using seismic interferometry. We then examine mechanisms of the measured velocity perturbation to investigate the presence of fluid-related anomalies in the subsurface seismic velocity changes. We compare the measured velocity perturbations to the peak ground velocity (PGV), peak ground acceleration (PGA), and modeled velocity changes due to coseismic static stress changes. Through these comparisons, we discuss the presence of an anomalous coseismic velocity drop, which potentially indicates the presence of an anomalous amount of subsurface fluids.

## Data and methods

### Measurement of coseismic relative velocity perturbations

Ambient noise seismic interferometry requires a stable noise source over time to derive genuine seismic velocity perturbations. We first investigated the frequency band of stable seismic noise in the Noto Peninsula to assess the feasibility of relative velocity perturbation measurements. For this purpose, we used the continuous waveform of Hi-net operated by the National Research Institute for Earth Science and Disaster Resilience (NIED 2019). The Hi-net stations are equipped with short-period three-component velocimeters with a natural frequency of 1 Hz, located in boreholes at a depth of more than 100 m (Obara et al. 2005). We computed the Power Spectral Densities (PSD) of the vertical component of Hi-net station YGDH (Fig. 1a), from 1 August 2023 to 31 December 2023 and corrected them for the high noise model from Peterson (1993) to find the frequency bands with particularly high noise with respect to the other frequencies. The obtained PSD reveal that the noise’s residual amplitude is particularly large and fairly steady over time between 0.3 and 1.1 Hz (Figure S1a). This suggests the presence of a strong and persistent noise source in this frequency band, which is suitable for the interferometry analysis in this region. The correlograms in the associated frequency band reveal that the daily correlations are stable over time (Fig. 1c); hence, they can be used to derive seismic velocity changes properly. We found that the noise PSD amplitude at site YGDH and the sea wave height recorded at Wajima station of Nationwide Ocean Wave information network for Ports and HArbourS (NOWPHAS) (Figure S1b) correlate very well in the frequency band of wind-driven swell (Longuet-Higgins and Jeffreys 1950; Gimbert and Tsai 2015). Therefore, the primary noise source we used for the velocity change measurement is likely generated by wind-driven swells in the Japan Sea. We explain the computation of the daily correlation functions and how we compared the PSD amplitude to the wave height in the supplementary material.

**Fig. 2:**
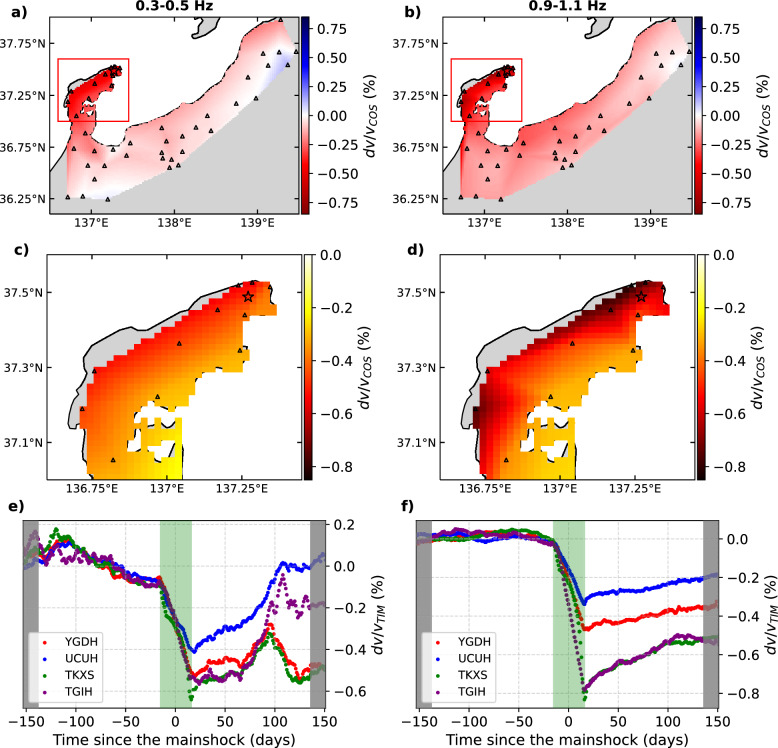
**a**, **b** Observed coseismic velocity perturbation averaged at each station $dv/v_{COS}$ in different frequency bands as labeled. The triangles show the location of the stations. Velocity perturbations between the stations are linearly interpolated. The open star indicates the location of the mainshock. The red rectangle in **a** and **b** indicates the region in **c** and **d**, respectively. See Figure S8 for maps in the intermediate frequency bands. **e**, **f** $dv/v_{TIM}$ time series from which $dv/v_{COS}$ is derived at the sites indicated in the legend. The periods during which correlations were averaged over less than 31 days are shaded in gray. The $dv/v_{TIM}$ during the period shaded in green are computed across the mainshock time (31-day moving average window)

To measure the coseismic relative seismic velocity perturbations in the Noto Peninsula, we adopted Hi-net stations that experienced Peak Ground Velocity (PGV) of > 5 cm/s (Fig. 1b) derived by USGS (United states geological survey earthquake shakemap nd). We omitted the rest of the stations below this PGV threshold because the coseismic velocity perturbation is expected to be very small there (Brenguier et al. 2014). To improve the spatial resolution of the observed coseismic velocity perturbations in the swarm zone, we included data from four temporary seismic stations (Fig. 1a). Each two of them were deployed by the Earthquake Research Institute (ERI), the University of Tokyo and Tohoku University (Sakai et al. 2022; Okada et al. 2024a, b). Each station is equipped with a short-period (1 and 2 Hz natural frequency for the ERI and Tohoku University stations, respectively) three-component seismometer, and their records are digitalized with a 100 Hz sampling frequency. A station TK2S, deployed by Tohoku University, was slightly moved by 14 ms away on 14 November 2023 with its new site name TK3S. In our analysis, we considered TK2S and TK3S as the same single station and renamed them TKXS (Fig. 1a). We deconvolved the time series of each station from the instrumental response and downsampled it to 25 Hz before performing our analysis. We computed the daily correlations from 1 August 2023 to 1 June 2024 between all components for all station pairs closer than 30 km from each other. For each station pair and component combination, we performed the cross-wavelet transform (Mao et al. 2019) to derive *dv*/*v* time series from coda waves. The cross-wavelet transform correlates the continuous wavelet transforms of two signals in the time-frequency domain, capturing their shared power and revealing the phase difference between them at each frequency and lag time. We focused on coda waves during a manually selected time window of 10–20 s starting from the arrival time of the ballistic surface waves, which we refer to as early coda waves. After this time window, the energy and coherence of the coda waves decay rapidly with lag time (Figures S2 d–e), preventing accurate phase difference measurements. Therefore, later coda waves were not regarded in our analysis. Further details of this time window selection workflow are provided in the supplementary material. Here, we employed the selected early coda waves by using the stack of all daily correlations of the pair and component combination as the reference. We label these time series $dv/v_{tim}$ (see List of abbreviations for the notation of different *dv*/*v* quantities derived in this study). Then, at each site, we averaged all $dv/v_{tim}$ time series in which the station is involved, leading to a single $dv/v_{TIM}$ time series for each site (Figure S3). We used $dv/v_{TIM}$ to extract the coseismic velocity change at each station, $dv/v_{COS}$ (Fig. 2, see supplementary material for the derivation of $dv/v_{COS}$).

We measured $dv/v_{tim}$, $dv/v_{TIM}$ and $dv/v_{COS}$ in four frequency bands: 0.3$-$0.5 Hz, 0.5$-$0.7 Hz, 0.7$-$0.9 Hz, and 0.9$-$1.1 Hz to investigate how seismic velocity changes at different depths. Such a depth-dependent analysis is made possible because the early coda waves used to measure seismic velocity changes result from the scattering of surface waves, which have frequency-dependent depth sensitivity. Assuming that the depth sensitivity of ballistic surface waves provides a reasonable approximation of the representative depth of our measurements, at 1.0 Hz and 0.4 Hz, the derived $dv/v_{COS}$ in the Noto peninsula are sensitive down to 0.5 km and 2.5 km depth, respectively, inferred from surface waves sensitivity kernels (Figure S6). We provided technical details on the estimation of seismic velocity changes and the calculation of sensitivity kernels in the supplementary material.

### Modeling of relative velocity changes due to coseismic static stress change

The relative velocity perturbation induced by static stress change is generally assumed to be linear to the volumetric elastic stress change (Niu et al. 2008; Wang et al. 2019; Sheng et al. 2024). We modeled coseismic static stress change using the slip model of Ma et al. (2024). We computed an elastic stress tensor distribution in the isotropic homogeneous half-space (Okada 1992) using the python package elastic_stress_py (kmaterna and Wong 2023). Here, we assumed a rigidity of 30 GPa and a Poisson’s ratio of 0.25. Then, we converted the coseismic volumetric stress change to the static relative velocity change *dv*/*v* by assuming exponential decay of the velocity-stress sensitivity with depth (Silver et al. 2007; Sheng et al. 2024):

1 $$\begin{aligned} \frac{dv}{v} = - C_n \times e^{- kd} \times \frac{\sigma _1+\sigma _2+\sigma _3}{3}, \end{aligned}$$

where $C_{n}$ denotes the velocity-stress sensitivity at the Earth’s surface, *k* is a decay rate, *d* is depth in km, and $\sigma _{1,2,3}$ are the principal stresses in MPa. We chose a decay rate *k* of 0.05 $\hbox {km}^{-1}$ from Sheng et al. (2024), and the sensitivity at the surface $C_n = 5 \times 10^{-9}$ $\hbox {Pa}^{-1}$ from Sano et al. (1999). A regional estimate of velocity-stress sensitivity ($\sim 8 \times 10^{-8}$ $\hbox {Pa}^{-1}$ with a bulk modulus *K* of 50 GPa) for the Noto Peninsula is available (Takano and Nishida 2023), but both sensitivity values would lead to estimations much larger than the observed coseismic velocity change as we show later (Sect. 3.2). With this, we decided to discuss only the spatial pattern of the observed and predicted velocity changes, and therefore, we prefer to adopt Sano et al. (1999)’s coefficient.

The measured coseismic velocity changes $dv/v_{COS}$ represent an integration of velocity variations across different depths. To account for the depth sensitivity of $dv/v_{COS}$ in the modeled static velocity drop, for each different frequency band (0.3$-$0.5, 0.5$-$0.7, 0.7$-$0.9, and 0.9$-$1.1 Hz), we regarded the depth sensitivity kernels of surface waves (0.4, 0.6, 0.8, 1.0 Hz) as the depth sensitivity profile of the measured velocity changes (Figure S6). We integrated the modeled static stress-induced velocity perturbations along the corresponding sensitivity kernels and obtained 2D velocity maps of $dv/v_{sta}$ (Figure S7). Finally, because the observed velocity perturbations are also inherently a spatial average of the velocity perturbations around the seismic stations (Pacheco and Snieder 2005), we spatially averaged the computed relative velocity change $dv/v_{sta}$ to make it directly comparable to $dv/v_{COS}$. When $dv/v_{COS}$ at station A, was derived from 6 cross-component single-station correlations and 18 cross-correlations with station B, the calculated static velocity perturbation $dv/v_{STA}$ at station A is the weighted average across $dv/v_{sta}$ at station A and the average $dv/v_{sta}$ along the direct path between stations A and B, with their weights to be 6 and 18.

## Results

### Coseismic velocity changes and their stability

**Fig. 3:**
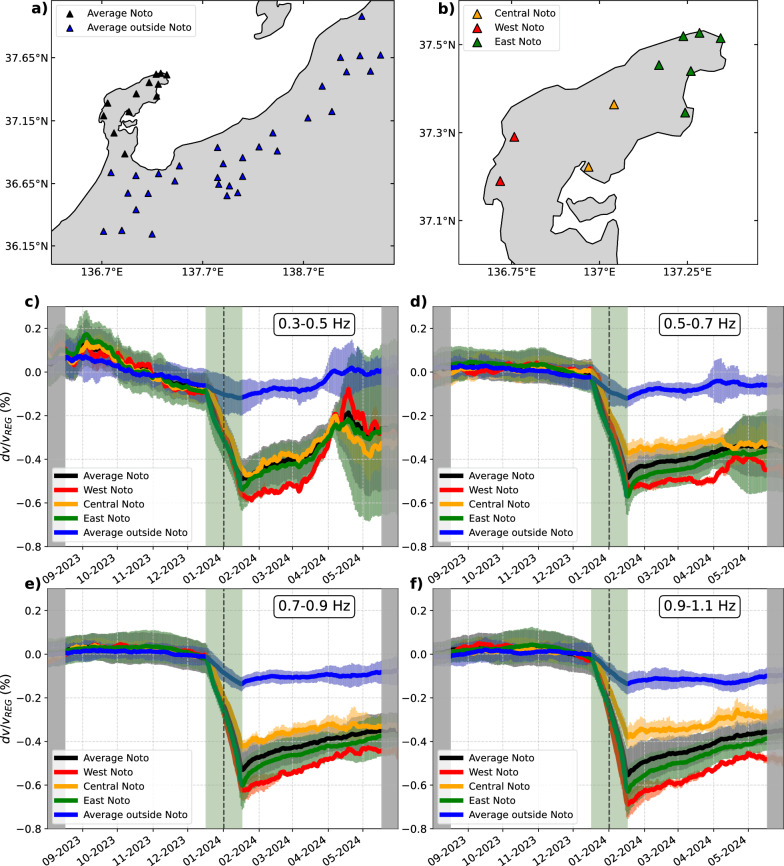
**a** and **b** Map of the stations over which the $dv/v_{REG}$ time series are averaged. All the stations are Hi-net, except for those labeled in Fig. 1a. **c**, **d**, **e**, **f** Regionally averaged $dv/v_{TIM}$ time series in different frequency bands as labeled, $dv/v_{REG}$. Error bars represent the standard deviation between the $dv/v_{TIM}$ time series averaged in a region. The stations used for averaging are shown in **a**–**b** with the corresponding color. The daily correlations were smoothed with a moving average (31-day window). The periods during which correlations were averaged over less than 31 days are shaded in gray. The $dv/v_{REG}$ during the period shaded in green are computed across the mainshock time (vertical black dashed line), and therefore they contain the rapid coseismic change

Overall, the measured coseismic velocity changes $dv/v_{COS}$ at each station, derived from $dv/v_{TIM}$ time series (Fig. 2e, f), is negative across all investigated frequency bands (Fig. 2a, b). The most significant velocity drop in $dv/v_{COS}$ occurs in the Noto Peninsula, with the average coseismic velocity decrease ranging from 0.42 to 0.53% between the 0.3–0.5 Hz and 0.9$-$1.1 Hz frequency bands, respectively (see site selection in Fig. 3a). Outside the peninsula, the average velocity drop is smaller, varying from 0.06% to 0.13% across the same frequency bands. Inside the peninsula, the largest coseismic velocity drops are recorded at sites closest to the mainshock fault segments (Fig. 2c, d, and Figure S8), with peak amplitudes ranging from 0.56 to 0.83% between 0.3$-$0.5 Hz and 0.9$-$1.1 Hz. The amplitude of the velocity drops decreases smoothly away from the fault segments at lower frequencies, whereas, at higher frequencies, it decreases more abruptly as the measured velocity perturbations along the coastline are significantly larger than those in the rest of the peninsula.

The coseismic velocity perturbation is very clear at most sites as seen in $dv/v_{TIM}$ (Figs. 2e, f, S3), but the $dv/v_{TIM}$ time series exhibit perturbations also before and after the mainshock, which could represent the temporal instability of our velocity measurement method. To illustrate regional perturbations outside the mainshock time, we performed multi-station stacking of $dv/v_{TIM}$ for different regions: outside and inside the peninsula (Fig. 3a) as well as the East, Central, and West parts of the peninsula, as subsets of sites inside the peninsula (Fig. 3b). The spatially averaged time series $dv/v_{REG}$ should more clearly illustrate other physical processes by mitigating random fluctuations (Fig. 3c–f). We identified a few velocity perturbations outside the mainshock time. For example, in the lower frequency band we analyzed (Fig. 3c), $dv/v_{REG}$ increased from August to September and then decreased until the mainshock. Following the mainshock, the gradual recovery of the seismic velocities is evident, representing the postseismic healing of the mainshock-induced velocity drop (Brenguier et al. 2008; Okubo et al. 2024). During this postseismic phase, a clear velocity drop was observed in April 2024 in all regions inside the peninsula at low frequencies (Fig. 3c), but not at higher frequencies (Fig. 3d–f). Nevertheless, the coseismic velocity drop is dominant at all the frequencies during our measurement period. We later discuss the impact of these transient perturbations outside the mainshock time on our $dv/v_{COS}$ measurements. In the following sections, we investigate the relationship between the observed velocity perturbation $dv/v_{COS}$, the modeled static stress-induced velocity change $dv/v_{STA}$, and PGV/PGA to explore the contribution of static and dynamic coseismic stress changes to the measured velocity drop.

### Effect of static stress change to the coseismic velocity drop

**Fig. 4:**
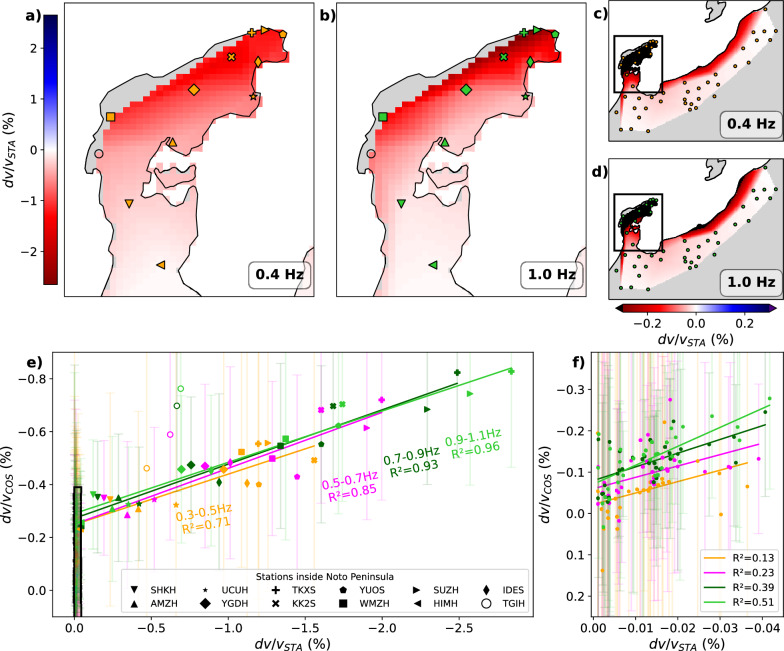
**a** Spatially integrated modeled coseismic static-stress-change-induced velocity perturbation $dv/v_{STA}$ at 0.4 Hz, using the coseismic slip model of Ma et al. (2024). The modeled velocity changes $dv/v_{sta}$ were integrated along the correlation paths (Figure S7) to match the resolution of our observed coseismic velocity changes $dv/v_{COS}$. The markers indicate the Hi-net and temporary station location, with their color indicating the frequency band of $dv/v_{COS}$ and $dv/v_{STA}$ in **e**. The station names for each marker are shown in **e**. The velocity changes between the stations are linearly interpolated. b same as **a** but at 1.0 Hz. **c** and **d** Broad view of the spatially integrated modeled velocity perturbations $dv/v_{STA}$. The black square in **c** and **d** indicates the region in **a** and **b**, respectively. **e** Comparison between $dv/v_{STA}$ and $dv/v_{COS}$ at all sites. Error bars represent the standard deviation of $dv/v_{COS}$ by propagating the error of the pre- and post-mainshock $dv/v_{TIM}$ measurement. Lines show the linear regression across all stations within the Noto Peninsula, excluding TGIH. **f** Zoom-in of the black rectangle in **e**). The lines represent the fit applied solely to sites inside the rectangle, corresponding to stations outside the Noto Peninsula

We compare the observed coseismic relative velocity changes $dv/v_{COS}$ (Fig. 2) to the modeled static stress-induced velocity changes $dv/v_{STA}$ (Fig. 4). Here, both the observed and modeled static relative velocity perturbations are consistently negative in the peninsula (Fig. 4). However, the amplitude of the modeled static velocity perturbation $dv/v_{STA}$ is significantly larger than $dv/v_{COS}$ inside the Peninsula. We chose the smaller value of the velocity-stress sensitivity at the surface *Cn* between the available estimates (Sano et al. 1999; Takano and Nishida 2023), but this difference may originate from the large uncertainty in their estimates, as its value can vary by several orders of magnitude (Yamamura et al. 2003). Therefore, we do not discuss the absolute difference in amplitude between $dv/v_{COS}$ and $dv/v_{STA}$ but rather focus on the comparison of their spatial patterns. In the subsequent discussion, we consider the region outside the Noto Peninsula as the far field and the Noto Peninsula as the near field (blue and black stations, respectively, in Fig. 3a).

In the near field, except for station TGIH, $dv/v_{COS}$ and $dv/v_{STA}$ have a similar spatial pattern (Fig. 4e). Here, we assess their similarity using a determination coefficient $\text {R}^{2}=1-\frac{\sum _i (y_i-f_i)^2}{\sum _i (y_i - {\bar{y}})^2}$, where $y_i$, ${\bar{y}}$ are the observed and average of observed $dv/v_{COS}$ values, respectively, and $f_i$ is predicted $dv/v_{COS}$ values by the linear regression. The determination coefficient ranged from 0.71 to 0.96 across all the frequency bands in our case, close to 1, meaning that the correlation between $dv/v_{COS}$ and $dv/v_{STA}$ is high. The $dv/v_{COS}$ at station TGIH apparently deviates more largely from the trend line with higher frequency, meaning that the velocity drop anomaly at TGIH is larger at the shallower depth. On the other hand, there is no clear correlation between $dv/v_{COS}$ and $dv/v_{STA}$ in the far field (Fig. 4f) with $\text {R}^{2}$ values between 0.13 and 0.51. Considering that the static stress changes decay rapidly away from the peninsula, the low correlation suggests that static stress change does not contribute significantly to the velocity perturbation outside the peninsula.

### Effect of dynamic stress changes on the coseismic velocity drop

**Fig. 5:**
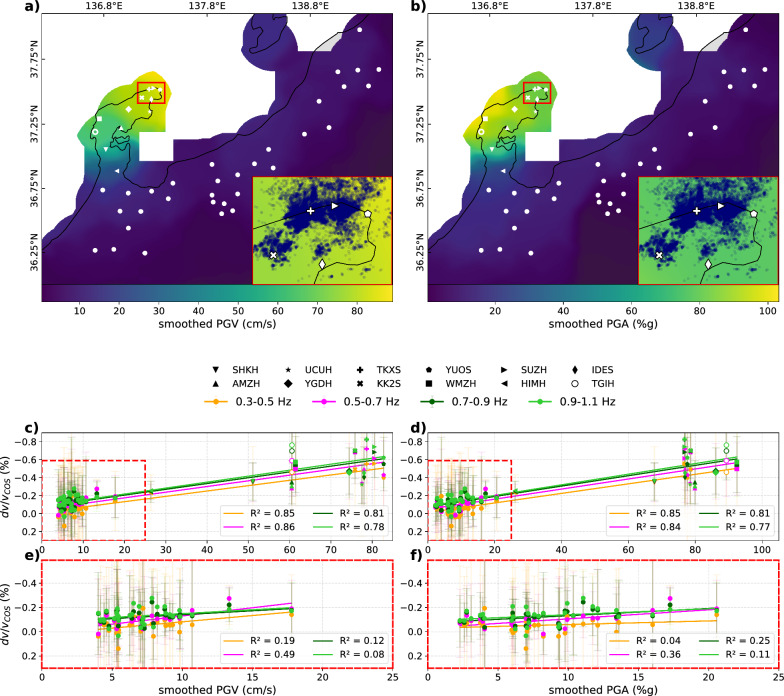
**a**, **b** Smoothed PGV (**a**) and PGA (**b**) maps derived from measured values by the USGS (Figure S10). White markers show the location of the Hi-net and the temporary sites. Red rectangles indicate the area of the insets. Navy dots in the insets represent the relocated hypocenters of regional seismicity before the 2024 mainshock (Fig. 1a). **c**–**d** The observed velocity drop $dv/v_{COS}$ as a function of smoothed PGV (**c**) and PGA (**d**), estimated at each site from smoothed maps (**a**–**b**). The lines represent linear regressions with $dv/v_{COS}$ from multiple frequency bands. Dots inside the red box correspond to the results of the far-field stations, located outside the peninsula, shown in **e**, **f**

We explore the relationship between dynamic stress changes and the observed coseismic velocity drop by using PGV and PGA values estimated at each station as proxies for dynamic stress changes (Brenguier et al. 2014; Wang et al. 2019). We employed PGV and PGA measurements spatially smoothed over 30 km, such that they have a roughly comparable spatial resolution to $dv/v_{COS}$ (see supplementary material for calculation of smoothed PGV/PGA). Both PGV and PGA exhibit similar first-order patterns, with amplitudes decreasing away from the peninsula (Fig. 5a, b), similar to the observed velocity change (Fig. 2a, b). However, their spatial distributions differ slightly inside the peninsula: PGV peaks at the northeastern tip near the epicenter, while PGA reaches its maximum at the western end. Both regions correspond to areas where the largest velocity drops are observed (Fig. 2c, d). Although we anticipate a non-linear relationship between the observed velocity drop and PGV/PGA in case of ground failure (Gomberg and Agnew 1996), the coseismic velocity perturbations $dv/v_{COS}$ and the smoothed PGV/PGA clearly correlate with each other with $\text {R}^{2}$ values ranging from 0.78 to 0.86 and 0.77 to 0.85, respectively, across all sites in both the near and far fields (Fig. 5c, d). The sites above the pre-mainshock swarm clusters (SUZH, TKXS, and KK2S, Fig. 5a, b) exhibit larger velocity drops than the rest of the sites at the tip of the peninsula (IDES and YUOS) although their smoothed PGV/PGA values are similar. In the far field, where the static stress change contribution is negligible, the correlation between the smoothed PGV/PGA and $dv/v_{COS}$ is also less clear than when using all sites, with lower $\text {R}^{2}$ values (Fig. 5e, f) because of the large uncertainties in $dv/v_{COS}$ and the small span of smoothed PGV/PGA values.

## Discussion

### Mechanisms causing the coseismic velocity perturbations

#### Near field

There is a large correlation between $dv/v_{COS}$ and $dv/v_{STA}$ at near-field sites in the peninsula, except for station TGIH, which highly deviates from the trend. The large correlation between $dv/v_{STA}$ and $dv/v_{COS}$ implies that they have similar spatial patterns (fit lines in Fig. 4e). The overall large shaking recorded in the entire peninsula (Fig. 5a, b) also showed their correlation with the observed velocity drop (Fig. 5c, d). However, in the near field, sites with larger PGV/PGA tend to also have larger $|dv/v_{STA}|$ (Figure S9). These results, therefore, indicate that we cannot isolate the contribution of each mechanism from our available coseismic velocity change analysis. The non-zero intercept of the linear fit between $dv/v_{STA}$ and $dv/v_{COS}$ in the near field and the clear postseismic velocity recovery (Fig. 3c–f) imply that the dynamic stress has a contribution to some extent to the observed velocity change. In contrast, it is difficult to prove that static stress changes impacted significantly the observed velocity changes. One way to quantitatively decipher the respective contribution of static and dynamic stress change to the coseismic velocity drops would be the continuous measurement of postseismic velocity recovery, as the effect of the latter will fully recover (Okubo et al. 2024). However, this recovery can take decades, so the quantitative evaluation of contributions from the static and dynamic stress change is beyond the scope of this study.

The large anomaly observed at TGIH (Fig. 4e) may also be explained by the effect of dynamic stress change. The smoothed PGA at this station is $\sim$ 97% g (Fig. 5b), but the original PGA measured at KiK-net station ISK006 colocating with TGIH is remarkably higher; the three-component average and the peak are $\sim$ 180% g (Figure S10 d) and $\sim$ 273% g (Figure S10b), respectively. This interpretation is also supported by the larger anomaly of the $dv/v_{COS}$ measurement with higher frequency measurement (Fig. 4e). These observations collectively suggest the existence of a shallow, strong, low relative-velocity anomaly, which could result from large dynamic-stress-induced subsurface damage (Wu et al. 2016). However, we cannot exclude the possibility that the anomaly at TGIH artificially originated from the modeled velocity change $dv/v_{STA}$ ignoring subsurface elastic heterogeneity (Okada 1992). The subsurface around station TGIH is likely more compliant than the rest of the peninsula, making it more prone to amplifying coseismic velocity changes, which is supported by the observation of secondary surface faulting with intense shallow aftershocks in the western end of the peninsula (Fukushima et al. 2024).

#### Far field

In the far field, the average amplitude of $dv/v_{STA}$ is about 0.015%, significantly smaller than the average amplitude of $dv/v_{COS}$, which is about 0.1% (Fig. 4f). In the near field where the static stress change’s contribution to the observed velocity changes must be largest, albeit unclear, the amplitude of $dv/v_{STA}$ is much larger than $dv/v_{COS}$, meaning that our $dv/v_{STA}$ is probably overestimated. Therefore, the amplitude of $dv/v_{STA}$ being much smaller than $dv/v_{COS}$ in the far field supports the hypothesis that the static stress change contribution to the observed velocity perturbation is negligible outside the peninsula. As the correlation between $dv/v_{COS}$ and smoothed PGV/PGA is large across the near and far fields (Fig. 5c-d) and the contribution of static stress change is negligible, it is likely that the coseismic velocity drop in the far field outside the Noto Peninsula is mostly dominated by dynamic stress change caused by the shaking of the earthquake.

#### Effect of fluids on coseismic seismic velocity changes in the swarm area

The sensitivity of seismic velocities to seismic shaking is increased in areas of high fluid pressure (Brenguier et al. 2014), which could be identified as anomalous large coseismic velocity drops. At the tip of the Noto Peninsula, the pre-mainshock seismic swarm activity is likely driven by upward fluid diffusion (Nishimura et al. 2023; Amezawa et al. 2023; Wang et al. 2024). With our approach, the coseismic velocity change is strongly impacted by the location of the nearby stations within a 30 km radius. When using solely Hi-net stations to derive $dv/v_{COS}$ at station SUZH, the estimated velocity change mostly accounts for paths away from the tip of the peninsula, as its neighboring Hi-net stations are outside the swarm zone and further away from the fault (Fig. 1a). Hence, the addition of temporary stations in the northeastern tip of the peninsula allowed us to increase the density of paths crossing this swarm zone, thus having a sufficient resolution of the measured velocity perturbation there (Figure S12). However, even though a larger velocity drop is observed in this region by adding the temporary sites, we do not identify any anomaly through the comparison of the spatial patterns of $dv/v_{COS}$ and $dv/v_{STA}$ (Fig. 4e). In other words, the stations above the pre-mainshock swarm clusters align similarly to the rest of the stations. A greater velocity decrease at the stations above the swarm than the others might not sufficiently support the presence of a velocity drop anomaly at the tip of the Noto Peninsula, considering the uncertainties of $dv/v_{COS}$ (Fig. 5). Therefore, by the time of the $M_w$ 7.5 occurrence, fluids as a possible driving mechanism of the pre-$M_w$ 7.5 swarm are unlikely to have reached the shallow subsurface (down to $\sim$ 2.5 km; Figure S6), where our seismic velocity perturbation measurement is sensitive.

### Other perturbation sources and their potential impact on coseismic velocity change

At low frequencies, $dv/v_{REG}$ exhibits clear perturbations outside the mainshock period that may bias the measured coseismic velocity changes (Fig. 3c). Prior to the mainshock, a large long-period velocity oscillation, characterized by an increase of the seismic velocities during summer and a decrease of the seismic velocities during winter, is evident. This seasonal velocity oscillation has been attributed to changes in snow and rainfall, air pressure, and sea level (Wang et al. 2017, 2024). After the mainshock, gradual recovery of coseismic velocity drop is observed, similar to other large earthquakes (Brenguier et al. 2008; Okubo et al. 2024; Kakiuchi et al. 2024). Since our estimation method of the coseismic velocity change does not take into account these effects, the obtained coseismic velocity change $dv/v_{COS}$ could be biased. Here, we employ another approach accounting for the seasonal oscillation and the postseismic recovery through a function fit (Hobiger et al. 2016) to verify the measured coseismic velocity change (see supplementary material). We call it a fit approach. Overall, we find good agreement between $dv/v_{COS}$ measured with the two different approaches, as they show similar spatial patterns (Figures S8 and S13). As a result, the relationship between $dv/v_{COS}$ and $dv/v_{STA}$ (Figure S14) or smoothed PGV/PGA (Figure S15) are also similar to those with the preferred method. This fit approach could be more powerful with measurements over a longer time, which will be useful to quantify the long-term velocity recovery properly (Okubo et al. 2024). Some discrepancies in the results with the two methods (Table S1 in supplementary material) are possibly due to the short time span of measurements and episodic velocity perturbations not accounted for (Fig. 3c).

## Conclusions

We investigated coseismic seismic velocity perturbations due to the 2024 Noto earthquake using data from both permanent Hi-net and temporary seismic stations. By measuring coseismic velocity changes ($dv/v_{COS}$) across multiple frequency bands, we observed a consistent velocity drop at all stations, with the largest drops closest to the mainshock slip peaks. We found a high correlation between the observed coseismic velocity perturbations and modeled velocity drop due to coseismic static stress changes ($dv/v_{STA}$) in the near field. The $dv/v_{COS}$ was also highly correlated with the smoothed PGV/PGA. However, due to similarities between the spatial pattern of PGV/PGA and $dv/v_{STA}$ and various assumptions and simplifications in our quantification of static and dynamic stress changes, their respective contribution to the coseismic velocity changes cannot be determined. While areas near the pre-mainshock swarm showed large velocity drops, these velocity drops were not anomalously larger than the rest of the sites, so we found no clear evidence of subsurface fluid-related anomalies in the velocity changes down to $\sim$ 2.5 km. In the far field, static stress changes were negligible, so the observed coseismic velocity drops were likely caused by the dynamic stress changes. The far-field velocity perturbations are still large (0.1% on average), so the Noto earthquake may offer a unique opportunity to explore the presence of pressurized fluids in volcanic areas in Central Japan (Brenguier et al. 2014), which may deserve a future investigation.

## Supplementary Information

Supplementary material 1.

## Data Availability

The Hi-net seismological data used in this study are archived at the Japanese National Research Institute for Earth Science and Disaster Prevention (NIED) (https://www.hinet.bosai.go.jp/?LANG=en and https://www.kyoshin.bosai.go.jp/) and are available upon request at the NIED data center. The data from Tohoku University supporting the findings of this study are available from Tomomi Okada upon request. The data from ERI supporting the findings of this study are available from Shinichi Sakai upon request.

## Abbreviations

PGV: Peak ground velocity
PGA: Peak ground acceleration
JMA: Japan Meteorological Agency
USGS: United States Geological Survey
Hi-net: High sensitivity seismograph network
K-net: Kyoshin Network
KiK-net: Kiban Kyoshin Network
ERI: Earthquake Research Institute
$dv/v_{tim}$: Time series of seismic relative velocity change, *dv*/*v*, for a given station pair and component combination, derived by comparing the daily (smoothed or not) correlations to the stack of all daily correlations.
$dv/v_{TIM}$: Spatial average of $dv/v_{tim}$ derived at each station from cross-component single-station correlations and cross-correlations in which it is involved. At each site, the inferred $dv/v_{TIM}$ is the mean between $dv/v_{tim}$ derived from all correlation pairs (and component combinations) involving the site.
$dv/v_{REG}$: Average of $dv/v_{TIM}$ time series in multiple regions.
$dv/v_{COS}$: Measured coseismic velocity change at each site from $dv/v_{TIM}$.
$dv/v_{sta}$: Modeled velocity perturbation induced by static stress change, integrated over depth with the sensitivity kernel of surface waves.
$dv/v_{STA}$: Spatial average of $dv/v_{sta}$ at each site. The averaging is done similarly to that of $dv/v_{TIM}$ from $dv/v_{tim}$. At each site, the inferred $dv/v_{STA}$ is the average between $dv/v_{sta}$ at the site location (for cross-component single-station correlations) and the average of $dv/v_{sta}$ along the direct path from the site to others (for cross-correlations), weighted by the number of $dv/v_{tim}$.
$\text {R}^{2}$: Coefficient of determination. The coefficient of determination measures how well a regression model explains the variance of a dependent variable based on independent variables. Ranging from 0 to 1, an $\text {R}^{2}$ of 1 indicates a perfect fit, while 0 means the model explains none of the variability in the data.
