# Supplementary material for: Coseismic crustal seismic velocity changes associated with the 2024 MW 7.5 Noto earthquake, Japan
Source: Earth Planets Space. 2025 Apr 21;77(1):51. doi: 10.1186/s40623-025-02177-x (PMC12011933; doi:10.1186/s40623-025-02177-x)
Supplement: Supplementary file 1 — Supplementary material 1. [file 40623_2025_2177_MOESM1_ESM.pdf]

Supporting Information for

Coseismic crustal seismic velocity changes associated with the 2024 M_w 7.5 Noto earthquake, Japan

Nicolas Paris *[1,2], Yuji Itoh[2], Florent Brenguier[3], Qing-Yu Wang[4,3], Yixiao Sheng[5], Tomomi Okada[6], Naoki Uchida[6], Quentin Higueret[3], Ryota Takagi[6], Shin'ichi Sakai[2], Satoshi Hirahara[6], and Shuutoku Kimura[6]

[1]Univ. Grenoble Alpes, CNRS, INRAE, IRD, Grenoble INP, IGE, Grenoble, France
[2]Earthquake Research Institute, The University of Tokyo, Japan
[3]ISTerre, University Grenoble Alpes, Grenoble, France
[4]ITES UMR 7063, Université de Strasbourg/CNRS, Strasbourg, France
[5]Laboratory of Seismology and Physics of the Earth's Interior, School of Earth and Space Sciences, University of Science and Technology of China, Hefei, China
[6]Research Center for Prediction of Earthquakes and Volcanic Eruptions, Graduate School of Science, Tohoku University, Sendai, Japan

March 13, 2025

*corresponding author

1 Processing of PSD and wave height time series

We computed the Power Spectral Densities (PSD) from the deconvolved time series of Hi-net station YGDH with windows of 1 hour and an overlap of 30 minutes. In order to identify a frequency band where the amplitude of the noise is abnormally strong, we corrected the observed PSD (PSD_{obs}) from the frequency-dependent high noise model, PSD_{NHMN} (Peterson, 1993) (Figure S1a). Then, to highlight the evolution of the residual PSD of the identified abnormally strong noise, we averaged it between 0.3-1.1 Hz (Figure S1b). We compared the average residual PSD time series to the wave height recorded by an ultrasonic wave height gauge of the National Ocean Wave information network for ports and HArborS (NOWPHAS) (available on `https://nowphas.mlit.go.jp/`) from 1 August 2023 to 31 December 2023. We applied a 5-hour long sliding median window to both residual PSD and wave height time series, to remove large amplitude spikes, and then smoothed the time series with an 8-hour long moving average window (Figure S1b).

2 Smoothed Peak Ground Velocity and Peak Ground Acceleration calculation

To gain spatially averaged Peak Ground Velocity and Acceleration (PGV and PGA, respectively) around the Hi-net and temporary sites, we used the PGV and PGA measured by the United States Geological Survey (USGS) using K-NET and KiK-net strong-motion seismographs (`https://earthquake.usgs.gov/earthquakes/eventpage/us6000m0xl/shakemap/metadata`). We first averaged the 3-component PGV and PGA values at each station to obtain a single estimate at each site (Figure S10c-d). We then mapped PGV and PGA by spatially averaging their measurements. At each grid point of the map, we computed the PGV and PGA as the median of the PGV and PGA from stations that are within a 30 km radius of each grid point. We finally smoothed the PGV and PGA maps with a Gaussian filter with a standard deviation of approximately 4 km.

3 Modeling of the sensitivity kernels and the depth integration of the modeled velocity change

We computed the phase velocity sensitivity kernels of Love and Rayleigh waves at 0.4, 0.6, 0.8 and 1.0 Hz with the python package disba (Luu, 2021) to determine the depth sensitivity of our measurements in the 0.3-0.5, 0.5-0.7, 0.7-0.9 and 0.9-1.1 Hz frequency bands, respectively. To model them, we use a velocity profile that is a combination of the JMA 2001 1D profile (`https://www.data.jma.go.jp/svd/eqev/data/bulletin/catalog/appendix/trtime/trt\_e.html`), and of the V4 3D structure model from (`https://www.j-shis.bosai.go.jp/map/`) to refine the velocity model in the subsurface. We averaged the velocity profile beneath each seismic station in the peninsula to derive a unique 1D profile from the V4 model. The final velocity profile uses the averaged V4 structure from the surface down to 500 m depth, and the JMA 2001 model from 500 m depth down to 10 km depth (Figure S5). Since the observed coseismic velocity changes were derived by averaging multiple component combinations, both Love and Rayleigh waves contribute to the measurements. For a given frequency, the actual sensitivity

kernel of the surface waves is therefore a linear combination of Rayleigh and Love waves sensitivity kernels (Figure S6).

We computed the velocity perturbations due to the coseismic static stress changes, dv/v_{sta}, by integrating the modeled velocity changes with the normalized sensitivity kernel of surface waves (Figure S7). We empirically found that the ratio of Rayleigh/Love waves sensitivity kernel used in this integration does not significantly impact the pattern of dv/v_{sta}, consequently we chose to calculate dv/v_{sta} using a sensitivity kernel composed of 100% Rayleigh waves.

4 Computation of daily correlations

Prior to the correlation calculation, we filtered the waveforms between 0.1 and 2.0 Hz and clipped them by 5 times the standard deviation to remove the effect of large amplitude sources. Then, we applied spectral whitening to the waveforms and finally, 1-bit normalized them. For each pair and component combination (with the exception of N-N, E-E and Z-Z for single-station correlations), we computed correlations in 30-minute windows with an overlap of 29 minutes, leading to 1410 correlations per day. We then normalized each of the 1410 correlations by their maximum amplitude, averaged them to make the daily correlation of a given station pair and component combination, and filtered them again in the 0.1-2.0 Hz band.

5 Determination of the optimal time window for interferometry

Ambient noise seismic interferometry determines a relative velocity perturbation by comparing an altered correlation to a reference one. In the resulting correlations, we found that early coda waves are stable over time, and can therefore be compared to derive relative velocity changes. To assess the optimal time window in the correlations to later derive reliable coseismic relative velocity changes for each pair, we first stacked daily correlations from 15 September 2023 to 31 December 2023 to make a reference correlation and from 02 January 2024 to 14 February 2024 to serve as an altered correlation function (Figure S2a-c). Before stacking daily correlations, we normalized them by their maximum amplitude. Then, for all component combinations, we measured the perturbation of the altered correlation function from the reference correlation using the cross-wavelet transform (Mao et al., 2019) with the python module by Qhig (2022). The cross-wavelet spectrum allows us to assess the coherence between two waveforms, as well as their lag shift dt in the lag and frequency domains. For each station pair, we averaged the coherence and lag shift panels across all component combinations (Figure S2). To better visualize the arrival time of the direct surface waves and the evolution of the coda waves' amplitude over lag time, for each component, we stacked all daily correlations from 1 August 2023 to 1 June 2024. Then, we computed the envelope of the stack with a Hilbert transform (then smoothed with a 2-second long moving average window), before averaging the envelopes from all component combinations (pink line in Figure S2d-g). Considering the frequency band where the residual noise amplitude is large, we averaged the lag shift panel (Figure S2f-g) from each component combination between 0.3-1.1 Hz, weighted by the corresponding coherence panel (Figure S2d-e). We then screened the correlograms, coherence panels and the dt time series of the pair in order to manually

find the optimum lag windows containing stable early coda waves. We repeated this process for all 148 pairs. During the screening process, we discarded station pairs and correlation sides that show weaker coherence (*e.g.* Figure S2d) or that contain clear glitches.

6 Computation of dv/v_{TIM}

For each station pair and component combination, we stacked all daily correlations from 1 August 2023 to 1 June 2024 normalized by their maximum amplitude to make the reference correlation. To obtain more stable daily correlations to compare to the reference, we averaged them with a 31-day moving average window. Then, for each pair and combination, we computed the cross-wavelet transform between the 31-day averaged daily correlations and the reference correlation. We averaged the dt measurements of the dt panel in the 4 chosen frequency bands, using the coherence as the weight, leading to 4 time series of dt as a function of the lag t per pair, combination, and day. Assuming uniform velocity perturbation along the ray path, the relative velocity change dv/v is linear to the dt/t Brenguier et al. (2008):

$$dv/v = -dt/t. \tag{S1}$$

Therefore, for each day, we then fitted a linear function to the $dt-t$ curve with the intercept constrained to 0 to gain the time series of relative velocity change, dv/v_{tim}, with Equation (S1). We computed the dv/v_{tim} for all non-discarded pairs, correlation sides, and all component combinations. As a result, we computed up to 6 dv/v_{tim} time series for each station pair in the case of single-station correlations (only cross-components) and up to 18 dv/v_{tim} time series in the case of cross-correlations. Then, for each station, the average velocity change time series dv/v_{TIM} is obtained by averaging the dv/v_{tim} time series from all component combinations and station pairs in which the station takes part of.

7 Estimation of dv/v_{COS}

At each station, we derived the coseismic velocity change dv/v_{COS} associated with the 2024 Noto earthquake from dv/v_{TIM}. We used two approaches to assess the coseismic velocity change; the first one being the preferred approach and the second being the fit approach, whose purpose is to verify the results of the first one.

7.1 Preferred approach

The first approach consists in measuring the difference in dv/v_{TIM} between the last day for which the 31-day averaged correlation does not include any daily correlation after the mainshock, and the first day for which the 31-day averaged correlation only includes daily correlations after the mainshock (see black crosses in Figure S3).

7.2 Fit approach

For the fit approach, we fitted the following function to dv/v_{TIM} at each station :

$$\hat{f}(t) = a + bH(t) + c \times \exp(-t/d)H(t) + e \times \sin(\omega t) + f \times \cos(\omega t), \tag{S2}$$

where each term corresponds to the constant offset of dv/v_{TIM}, the non-recovering coseismic step, the recovering coseismic drop with the recovery time constant d, and the seasonal seismic velocity variations with the frequency constant ω, respectively. t is the number of days since the mainshock, $H(t)$ is the Heaviside function, and ω, the seasonal term's frequency, is set to $2\pi/365$ days^{-1}. We determined all parameters including a, b, c, d, e, and f, by a non-linear least-squares fit to dv/v_{TIM} time series derived from daily (no 31-day averaging) correlations, minimizing:

$$\chi = \frac{\sum_{i=1}^{N}[1/(\sigma_{err}(t_i)]^2 \times [f(t_i) - \hat{f}(t_i)]^2}{\sum_{i=1}^{N}[1/(\sigma_{err}(t_i)]^2}, \tag{S3}$$

where $f(t)$ is the observed dv/v_{TIM}, $\hat{f}(t)$ is the associated fit, and $\sigma_{err}(t_i)$ is the standard deviation of dv/v_{tim} for all averaged component combinations and station pairs averaged to derive dv/v_{TIM} at time index i.

8 Summary of measured dv/v_{COS}

Table S1: Summary of dv/v_{COS} derived at sites inside and outside the Noto Peninsula.

	**Preferred Method**		**Fit**	
**Frequency Range (Hz)**	**Average** dv/v_{COS}	**Peak** dv/v_{COS}	**Average** dv/v_{COS}	**Peak** dv/v_{COS}
**Inside Peninsula**				
0.3-0.5	-0.42%	-0.56%	-0.46%	-0.62%
0.5-0.7	-0.48%	-0.72%	-0.47%	-0.77%
0.7-0.9	-0.51%	-0.82%	-0.45%	-0.66%
0.9-1.1	-0.53%	-0.83%	-0.41%	-0.66%
**Outside Peninsula**				
0.3-0.5	-0.06%	-0.22%	-0.12%	-0.26%
0.5-0.7	-0.10%	-0.28%	-0.14%	-0.28%
0.7-0.9	-0.12%	-0.25%	-0.16%	-0.63%
0.9-1.1	-0.13%	-0.28%	-0.11%	-0.27%

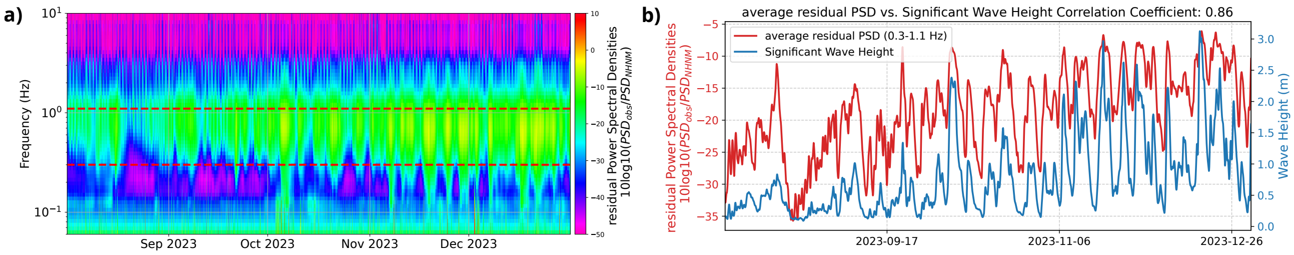

Figure S1: a) Observed Power Spectral Densities (PSD_{obs}) from 1 August 2023 to 31 December 2023 corrected for the high noise model (PSD_{NHMN}). b) Comparison between wave height at Wajima (blue) and average residual PSD from a) in 0.3-1.1 Hz frequency band at YGDH (red). See Figure 1a for the station location.

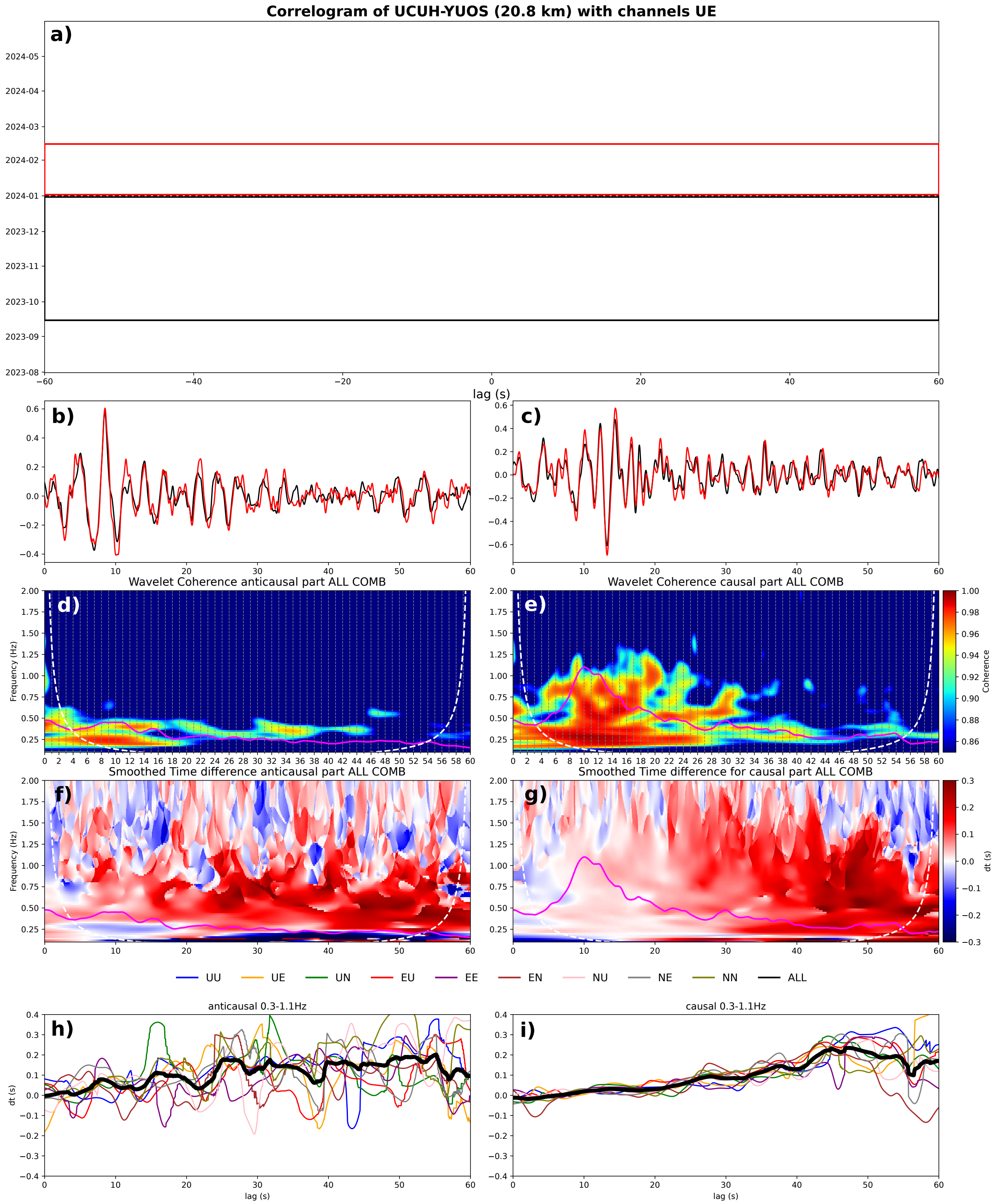

Figure S2: a) Correlogram of pair UCUH-YUOS for component combination UE filtered between 0.1-2.0Hz. The black and red windows correspond to the reference and altered correlation stack windows, respectively. b) and c) are the obtained stacked correlations in the anticausal and causal parts, respectively, using the windows in a). d) and e) are the average of coherence panels between the reference and altered correlation for all component combinations. f) and g) are the average lag shift panels, derived in the same way as d) and e). h) and i) are the resulting lag shift dt time series resulting from the averaging of f) and g) between 0.3-1.1 Hz, weighted by d) and e), respectively.

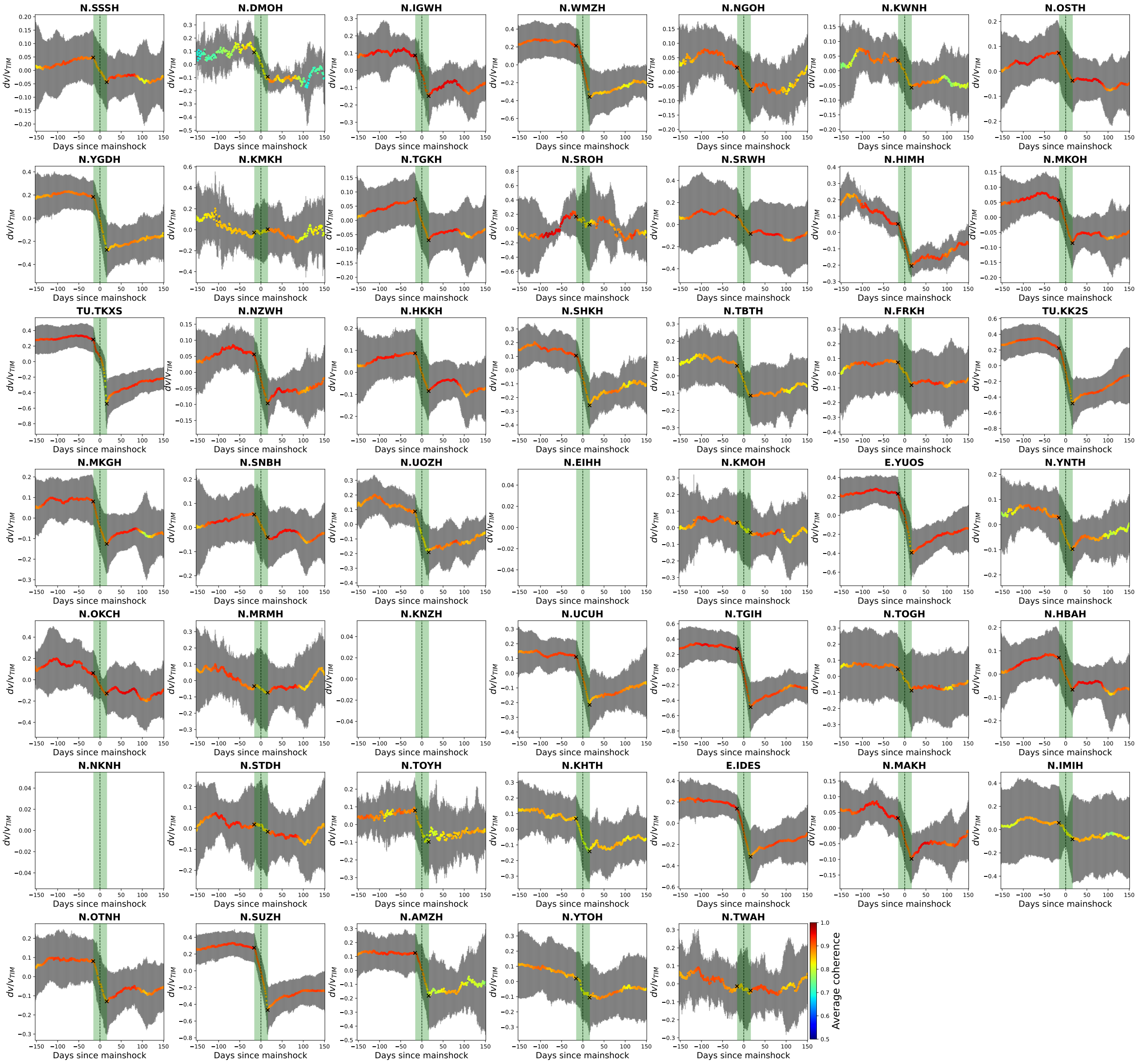

Figure S3: dv/v_{TIM} time series at each station in 0.9-1.1 Hz (dots with color) with the gray shade indicating the standard deviation of all dv/v_{tim} involving the station. The dot color indicates the average coherence between the daily correlation and the reference among the dv/v_{tim} of the given station. Black crosses indicate the days used to estimate the coseismic velocity change with the preferred approach. Stations location is shown in Figure S4.

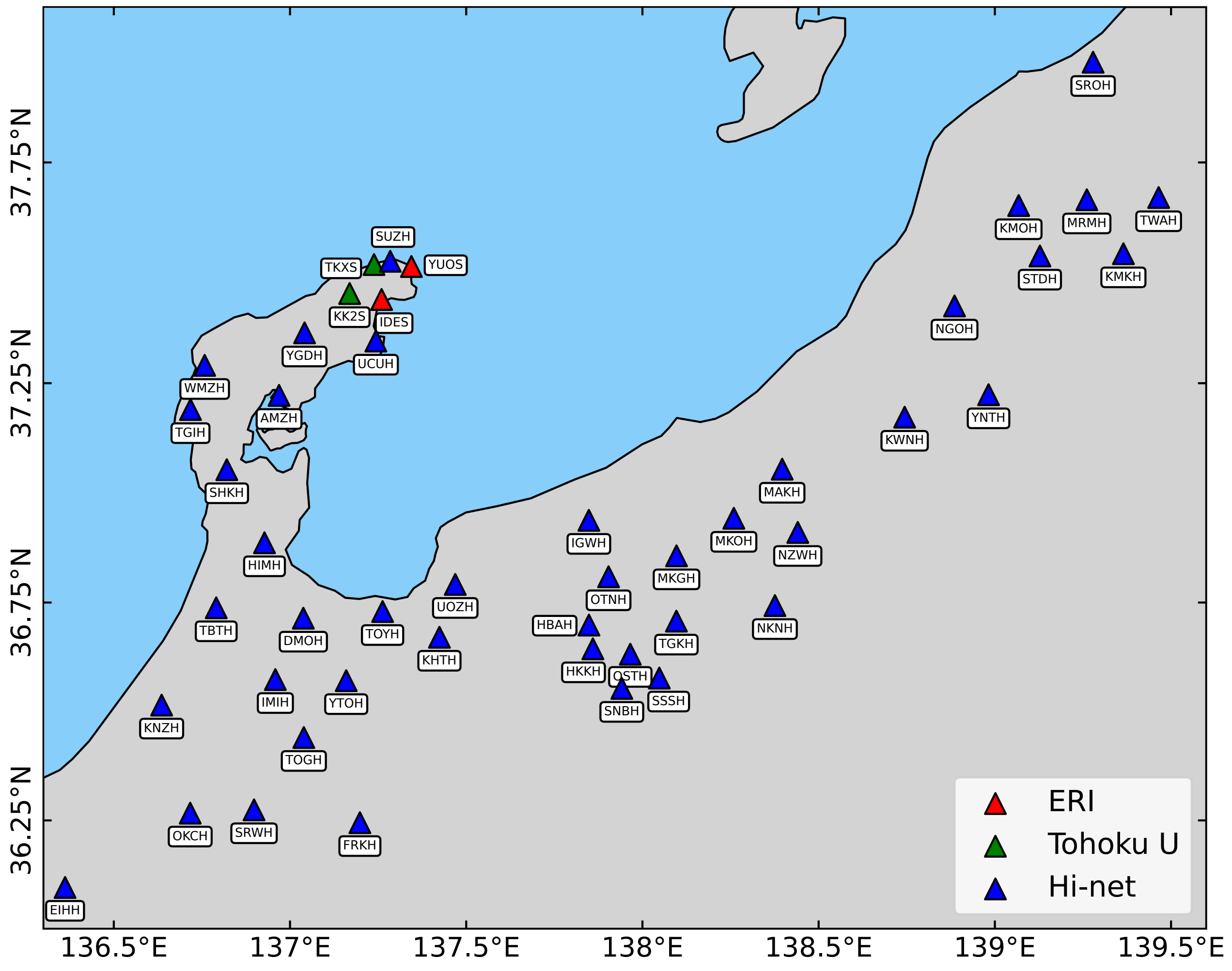

Figure S4: Station map. Triangles indicate the location of seismograms used in this study, with colors indicating different observers as labeled.

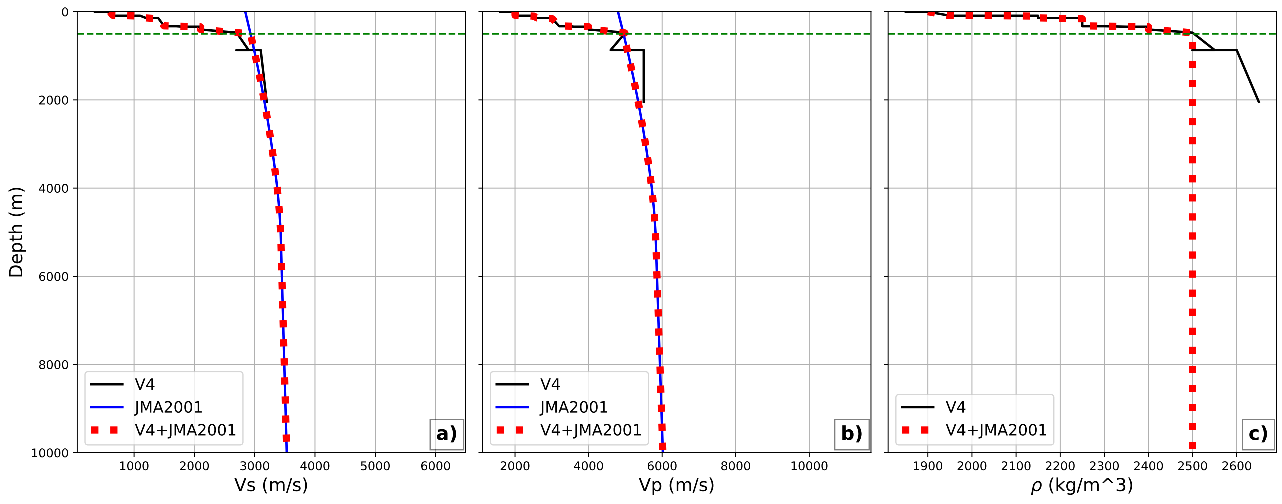

Figure S5: V_s (a), V_p (b) and density (c) profiles from JMA2001 and J-SHIS V4 models as labeled.

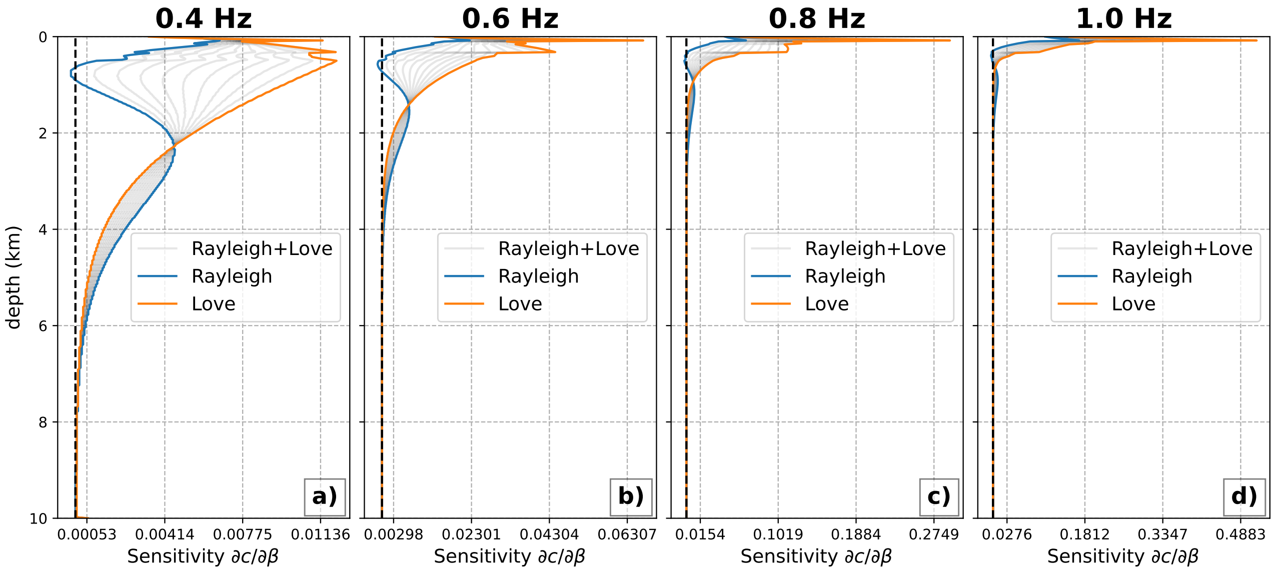

Figure S6: Sensitivity of surface waves phase velocity to S-wave velocity at 0.4 Hz (a), 0.6 Hz (b), 0.8 Hz (c) and 1.0 Hz (d). Blue, orange, and gray curves represent the sensitivity of Rayleigh, Love, and linearly combined Rayleigh and Love waves, respectively.

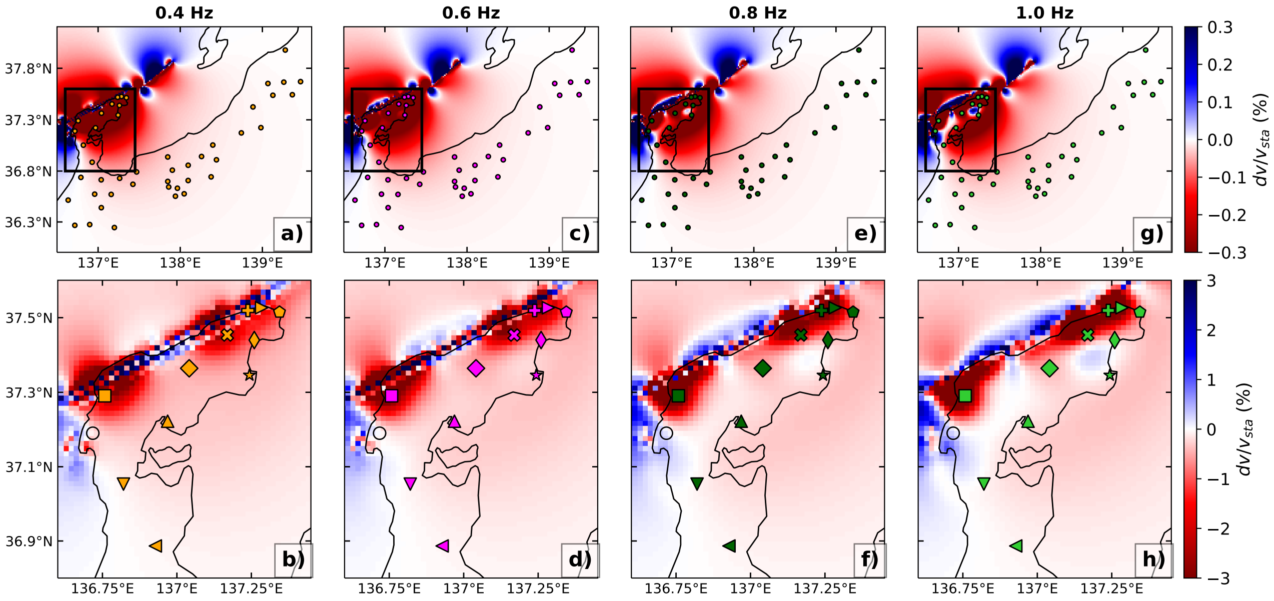

Figure S7: Velocity changes induced by static coseismic stress changes (dv/v_{sta}), derived by integrating the modeled depth-dependent static stress changes with surface wave depth sensitivity kernels (Figure S6). The coseismic slip model is after Ma et al. (2024). The markers indicate the location of Hi-net and temporary stations. The shape and color of the markers are for different stations and frequencies as shown in Figure 4.

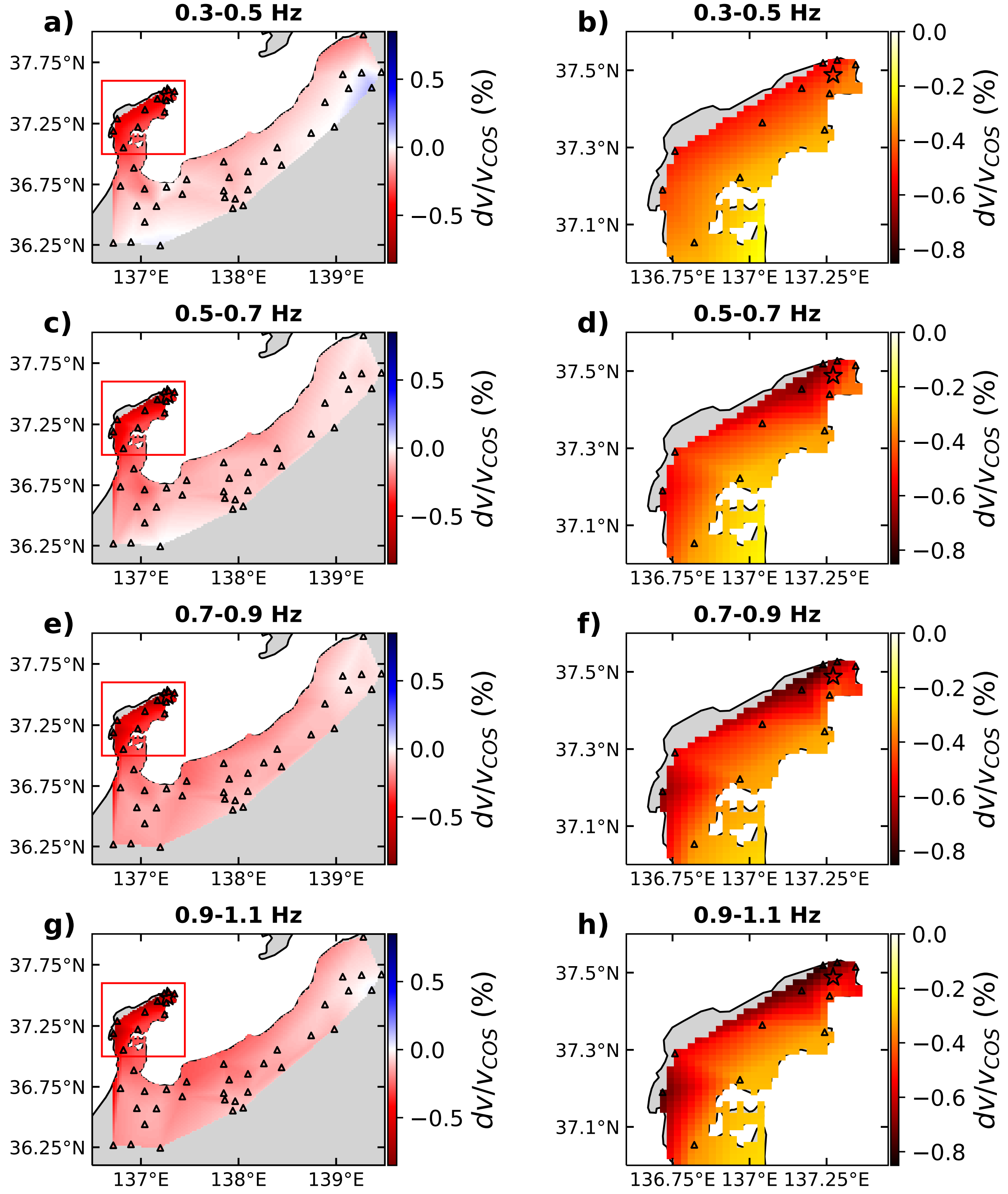

Figure S8: Observed coseismic velocity perturbation dv/v_{COS} with the preferred approach at different frequency bands as labeled. The panels a, b, g, and h are identical to Figures 2a, c, b, and d, respectively. Velocity perturbations between stations are linearly interpolated. The triangles show the location of the stations. The open star indicates the location of the mainshock. The red rectangle in the left panels indicates the region in the right panels.

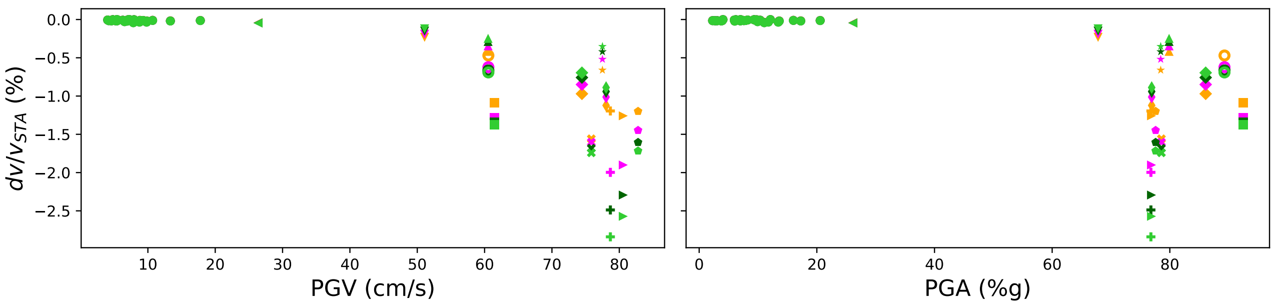

Figure S9: Comparison between dv/v_{STA} and PGV (a) / PGA (b). The color and shape of the markers indicate the frequency band station, respectively, as in figure 5.

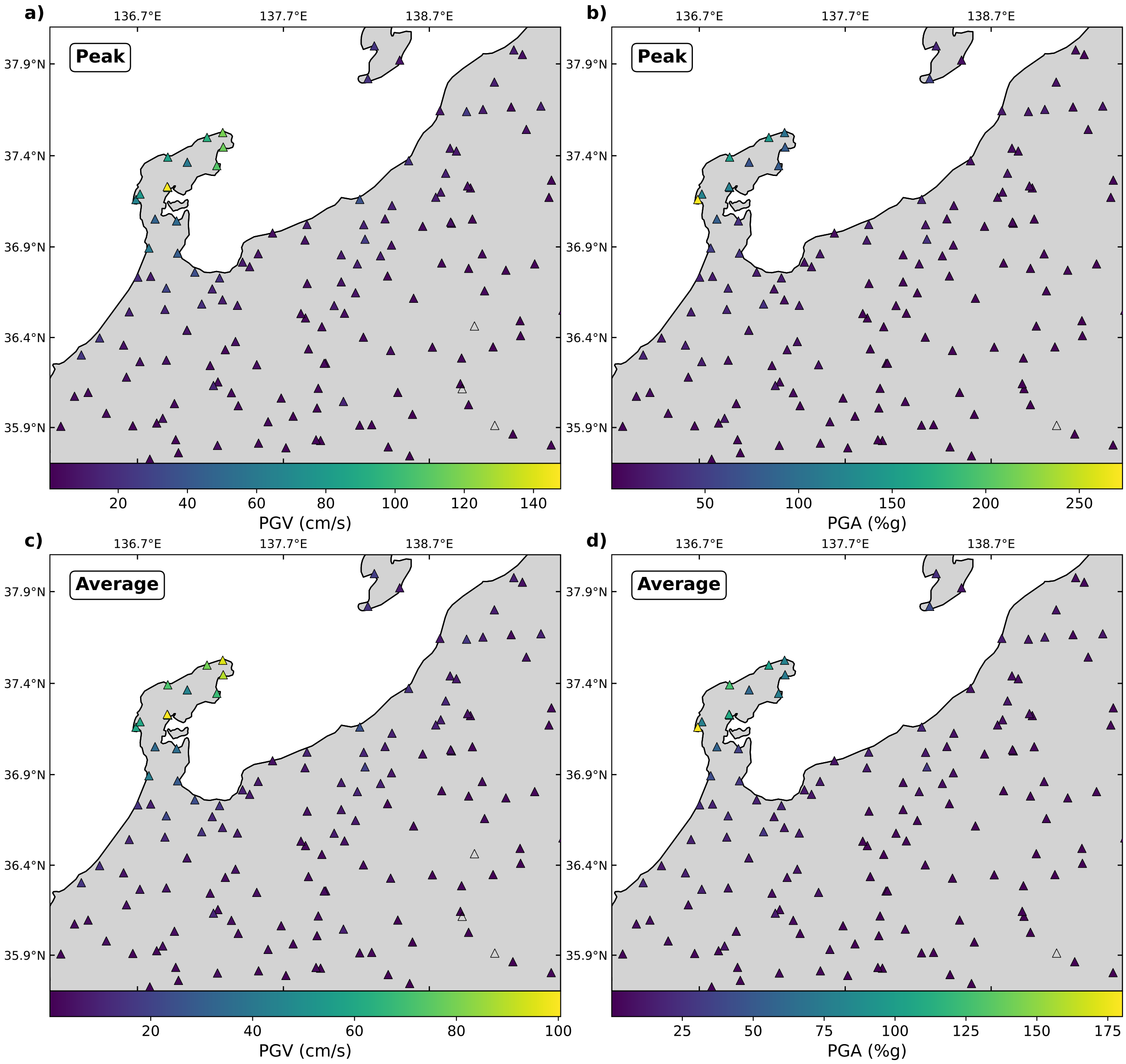

Figure S10: Measured peak PGV (a), and PGA (b) at KiK-net and K-net stations by USGS (`https://earthquake.usgs.gov/earthquakes/eventpage/us6000m0xl/shakemap/metadata`). Measured PGV (c), and PGA (d) averaged over the three components.

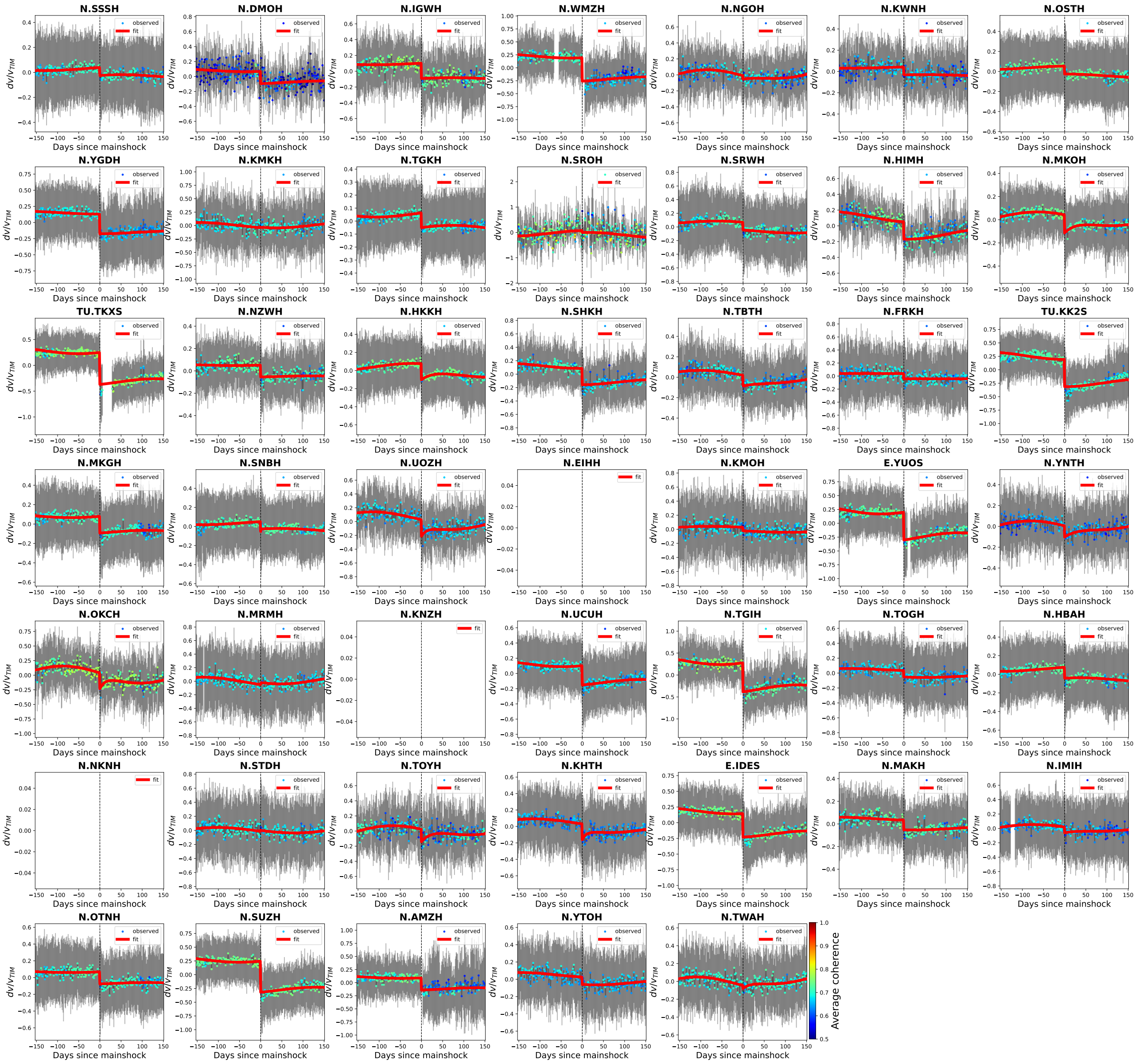

Figure S11: Same as Figure S3, except that daily correlations were not averaged with a 31-day moving average window. The red curve represents the fit used to derive dv/v_{COS} with the fit approach.

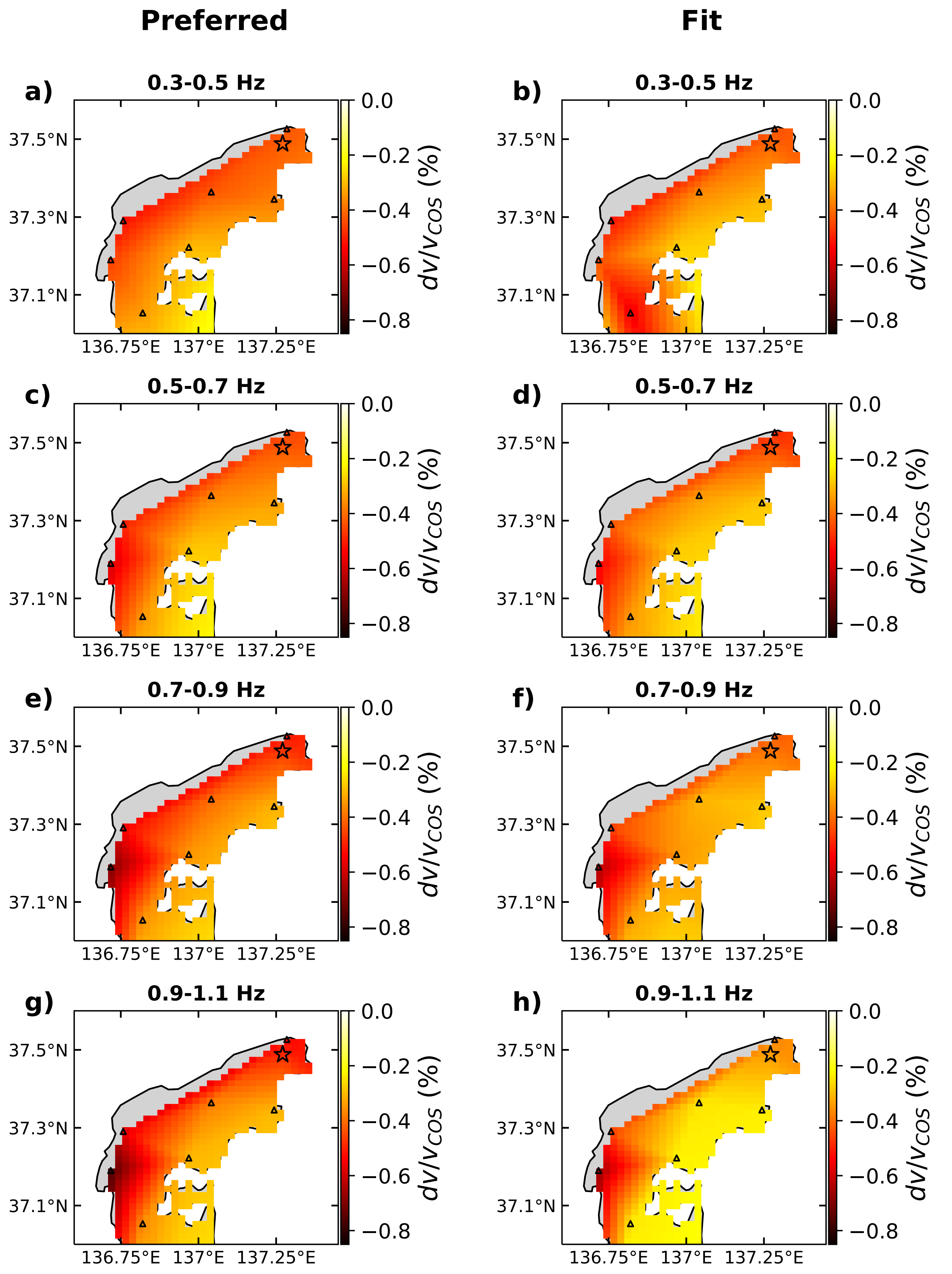

Figure S12: Coseismic velocity change in the peninsula derived using exclusively Hi-net stations. Panels a), c), e), and g) use the preferred method while panels b), d), f), and h) use the fit approach.

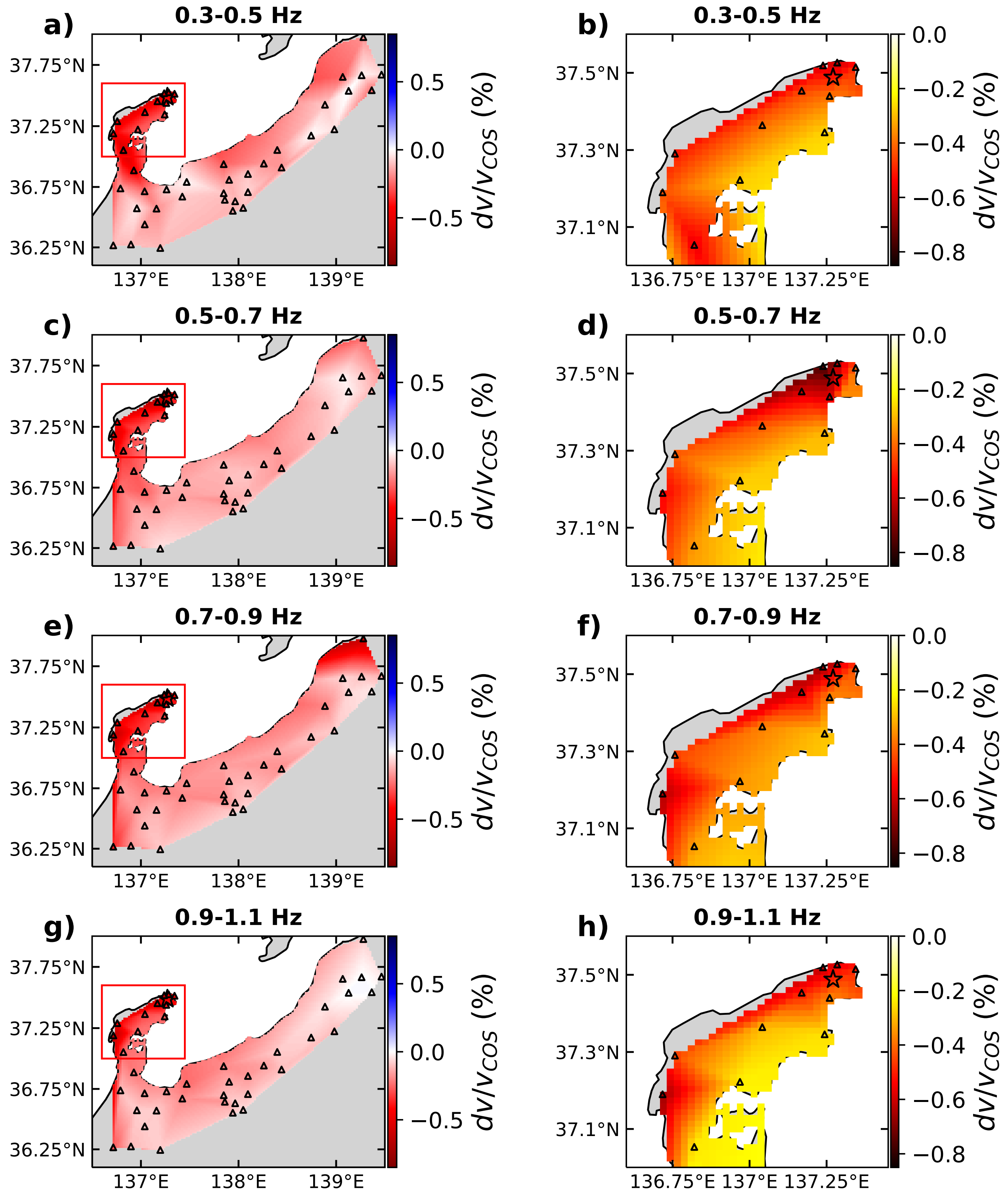

Figure S13: Same as Figure S8 but using the fit approach.

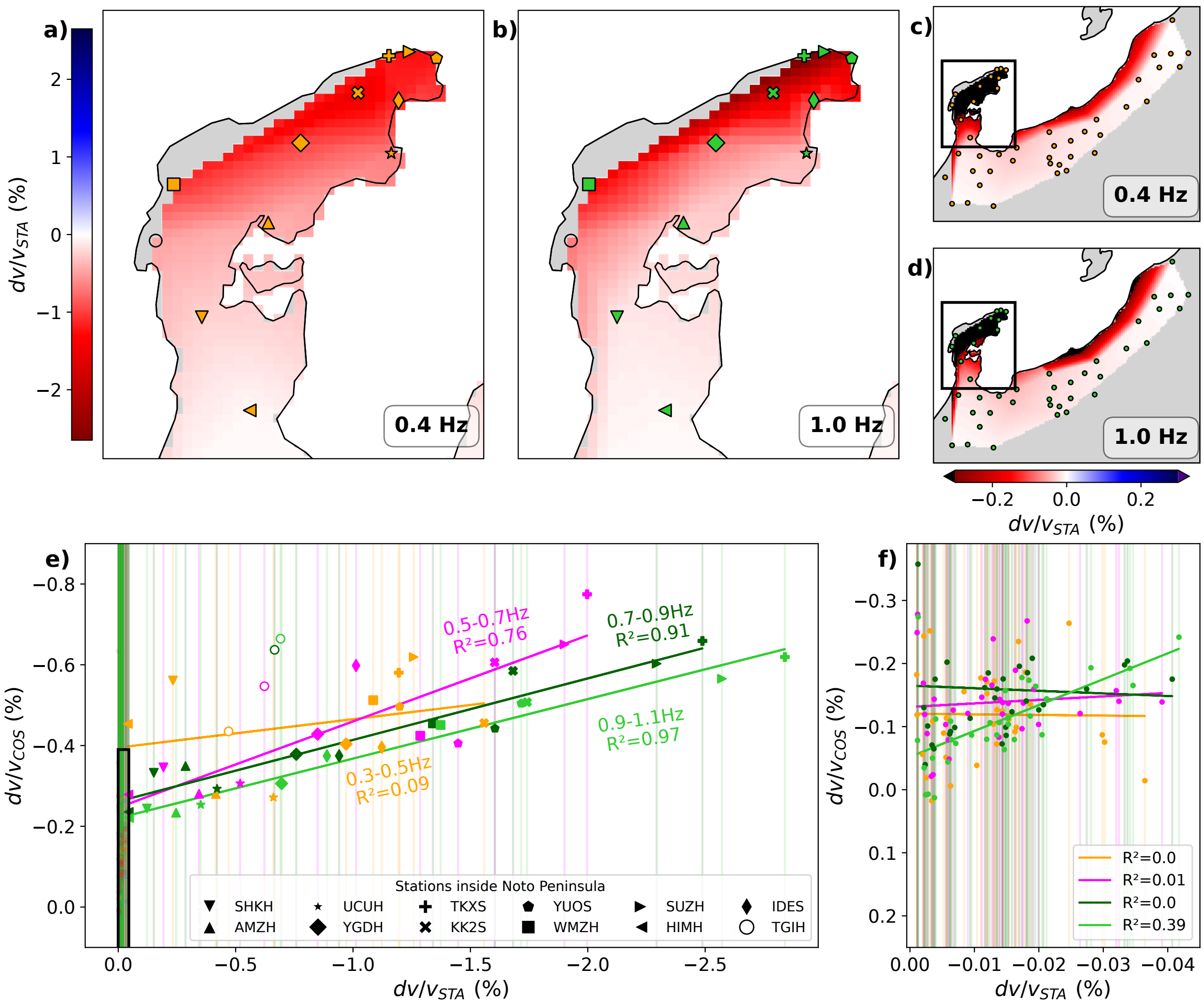

Figure S14: Same as Figure 4 but using the observed velocity changes dv/v_{COS} measured with the fit approach.

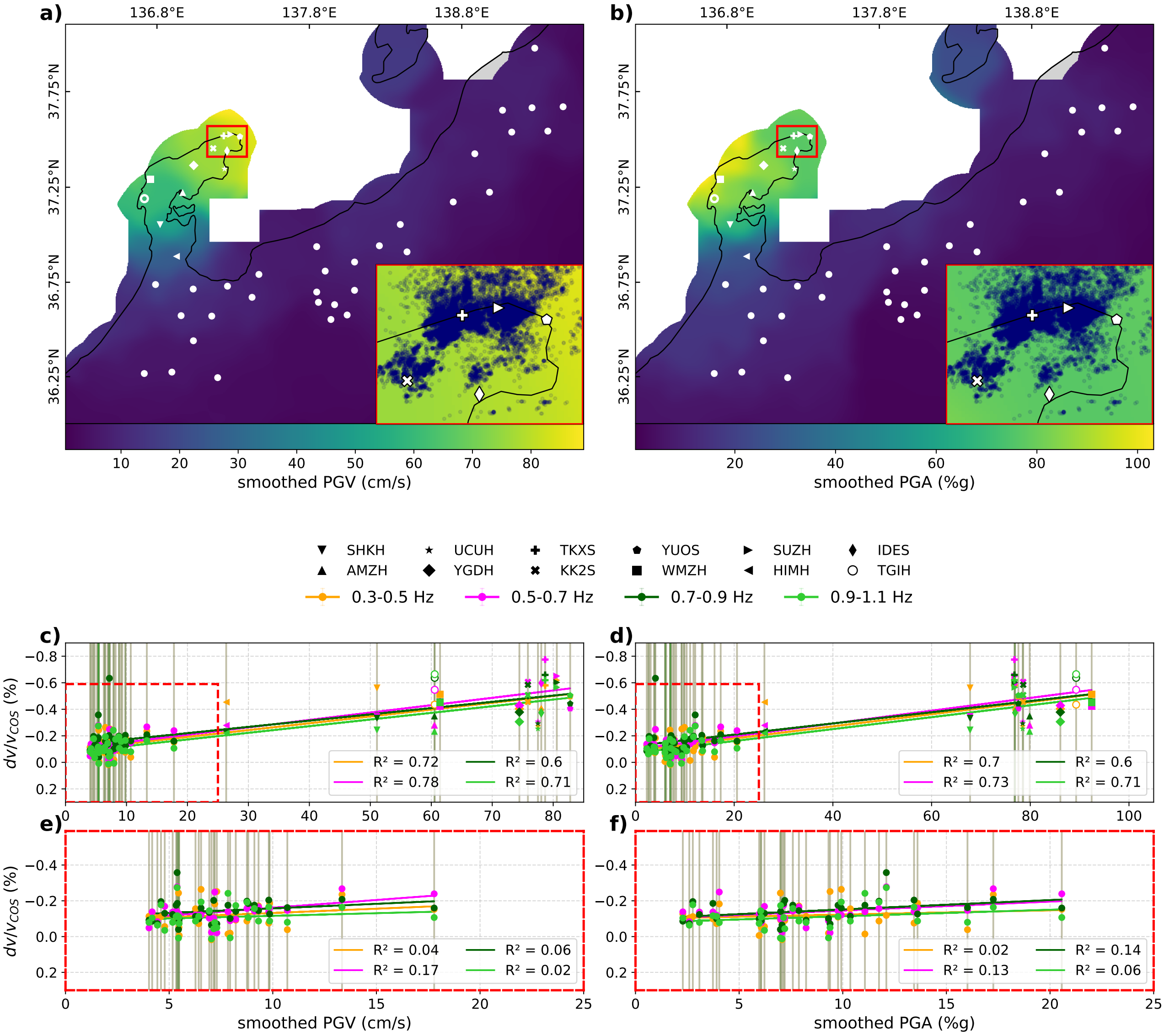

Figure S15: Same as Figure 5 but dv/v_{COS} using the fit approach.

References

Brenguier, F., Campillo, M., Hadziioannou, C., Shapiro, N. M., Nadeau, R. M., and Larose, E. (2008). Postseismic relaxation along the san andreas fault at parkfield from continuous seismological observations. *Science*, 321(5895):1478–1481.

Luu, K. (2021). disba: Numba-accelerated computation of surface wave dispersion. Zenodo. Version v0.6.1.

Ma, Z., Zeng, H., Luo, H., Liu, Z., Jiang, Y., Aoki, Y., Wang, W., Itoh, Y., Lyu, M., Cui, Y., Yun, S.-H., Hill, E. M., and Wei, S. (2024). Slow rupture in a fluid-rich fault zone initiated the 2024 M_w 7.5 noto earthquake. *Science*, 385(6711):866–871.

Mao, S., Mordret, A., Campillo, M., Fang, H., and van der Hilst, R. D. (2019). On the measurement of seismic traveltime changes in the time–frequency domain with wavelet cross-spectrum analysis. *Geophysical Journal International*, 221(1):550–568.

Peterson, J. R. (1993). Observations and modeling of seismic background noise. Technical report. Report.

Qhig (2022). Qhig/cross-wavelet-transform.
